# Recent Developments in Protein-Based Hydrogels for Advanced Drug Delivery Applications

**DOI:** 10.3390/pharmaceutics18010074

**Published:** 2026-01-06

**Authors:** Giuseppe Scopelliti, Claudia Ferraro, Ortensia Ilaria Parisi, Marco Dattilo

**Affiliations:** 1 Department of Pharmacy, Health and Nutritional Sciences, University of Calabria, 87036 Rende, CS, Italy; giuseppe.scopelliti@unical.it (G.S.); claudia.ferraro@unical.it (C.F.); marco.dattilo@unical.it (M.D.); 2 Macrofarm s.r.l., c/o Department of Pharmacy, Health and Nutritional Sciences, University of Calabria, 87036 Rende, CS, Italy

**Keywords:** protein, hydrogels, drug delivery, cancer therapy, natural polymers, gelatin, collagen, silk fibroin, soy protein, peptide-based hydrogels

## Abstract

Protein-based hydrogels are increasingly recognized as promising biomaterials for advanced drug delivery, owing to their biocompatibility, biodegradability, and ability to recreate extracellular matrix-like environments. By tailoring the protein source, crosslinking strategy, molecular architecture, and functionalization, these hydrogels can be engineered to mimic the mechanical and biological features of native tissues. Protein-derived hydrogels are currently explored across biomedical and pharmaceutical fields, including drug delivery systems, wound healing, tissue engineering, and, notably, cancer therapy. In recent years, growing attention has been directed toward natural protein hydrogels because of their inherent bioactivity and versatile physicochemical properties. This review provides an updated overview of protein-based hydrogel classification, properties, and fabrication methods. It highlights several widely studied natural proteins, such as gelatin, collagen, silk fibroin, soy protein, casein, and whey protein, that can form hydrogels through physical, chemical, or enzymatic crosslinking. These materials offer tunable mechanical behavior, controllable degradation rates, and abundant functional groups that support efficient drug loading and the development of stimuli-responsive platforms. Furthermore, we examine current advances in their application as drug delivery systems, with particular emphasis on cancer treatment. Protein-based hydrogels have demonstrated the ability to protect therapeutic molecules, provide sustained or targeted release, and enhance therapeutic effectiveness. Although critical challenges, such as batch-to-batch variability, sterilization-induced denaturation, and the requirement for comprehensive long-term immunogenicity assessment, must still be addressed to enable successful translation from preclinical studies to clinical application, ongoing advances in the design and functionalization of natural protein hydrogels highlight their promise as next-generation platforms for precision drug delivery.

## 1. Introduction

Polymeric hydrogels are three-dimensional (3D), crosslinked networks capable of absorbing large amounts of water and biological fluids while maintaining structural integrity [1]. Their elasticity and high water content give them tissue-like mechanical properties and excellent biocompatibility, making them highly suitable for biomedical applications [2]. Over the years, hydrogels have been used across diverse fields such as drug delivery, tissue engineering, wound healing, and cancer therapy due to their tunable chemical composition and porous architecture, which enable encapsulation and controlled release of therapeutic agents [3]. Hydrogels can be classified into conventional and stimuli-responsive (“smart”) types. Conventional hydrogels typically respond to environmental changes such as pH or temperature only through simple swelling or deswelling behavior and often suffer from poor mechanical strength [4]. Smart hydrogels, by contrast, are engineered to undergo significant physicochemical changes in response to stimuli such as pH, temperature, ionic strength, light, or magnetic fields. These responsive behaviors allow precise control over drug release kinetics, localization, and timing, making them highly attractive for on-demand therapy and precision medicine [5].

Among the various hydrogel systems, protein-based hydrogels have emerged as particularly promising biomaterials due to their inherent biocompatibility, biodegradability, and biological functionality. Derived from natural or recombinant proteins such as collagen, gelatin, silk fibroin [6], albumin, and sericin, these hydrogels can closely mimic the extracellular matrix (ECM), promoting cell adhesion, proliferation, and differentiation [7]. Their mechanical and biochemical tunability enables them to function as scaffolds for tissue regeneration and as vehicles for localized, sustained drug delivery [8]. Protein-based hydrogels offer several advantages over synthetic polymer networks. Their natural origin ensures minimal immunogenicity and excellent integration with biological tissues. Moreover, their molecular structure can be modified through chemical crosslinking, enzymatic reactions, or genetic engineering, allowing the creation of stimuli-responsive systems that react to pH, temperature, or enzymatic activity. Such “smart” protein hydrogels have been exploited in cancer therapy for localized drug delivery, reducing off-target toxicity and enhancing therapeutic efficacy [9]. In wound healing applications, protein-based hydrogels provide a moist, bioactive environment that supports angiogenesis and accelerates tissue repair, especially when loaded with growth factors or antimicrobial agents [10]. Similarly, in drug delivery, they allow controlled and prolonged release of therapeutic molecules while maintaining bioactivity and minimizing systemic side effects [11]. Advances in nanotechnology and hybrid material design have further enhanced their stability and mechanical strength, extending their use to regenerative medicine, cancer immunotherapy, and targeted combination therapies [12]. Given these advancements, protein-based hydrogels represent a versatile and powerful class of biomaterials that bridge the gap between natural and synthetic systems. Their tunable properties, multifunctionality, and biological compatibility make them ideal candidates for next-generation therapeutic platforms. This review explores the design principles, modification strategies, and biomedical applications of natural protein-based hydrogels, with a particular emphasis on their role in drug delivery and cancer therapy.

## 2. Materials and Methods

A comprehensive literature review was conducted utilizing PubMed and Scopus as primary search databases. The search was refined to include full-text, peer-reviewed research articles in English, focusing on protein-based hydrogels and their applications in drug delivery systems. To ensure relevance, only studies published between 2018 and 2025 were considered. The search strategy incorporated specific keywords, namely “hydrogel”, “protein”, “drug delivery system”, and “cancer treatment”, with query formulations tailored to each database. Relevant studies were systematically analyzed, and the extracted data were categorized into key thematic areas, including hydrogel classification, physicochemical properties, synthesis techniques, applications in drug delivery, and their role in cancer therapy.

## 3. Natural Polymer-Based Hydrogels

Naturally derived hydrogels are increasingly regarded as the most promising materials for pharmaceutical, biomedical, and cancer therapies due to their intrinsic biofunctionality. In pharmaceutical applications, natural hydrogels act as smart drug delivery platforms, capable of encapsulating bioactive molecules and releasing them in a controlled and sustained manner, thereby enhancing drug bioavailability and reducing systemic toxicity [13]. In biomedical fields, they are highly valued for their biomimetic properties, supporting cell adhesion, proliferation, and differentiation, which are crucial for wound healing, tissue engineering, and regenerative medicine. Moreover, their tunable swelling behavior, biodegradability, and mild gelation conditions enable the encapsulation of delicate biomolecules such as proteins, growth factors, and nucleic acids without loss of activity [14]. In cancer therapy, they play an increasingly important role in localized and stimuli-responsive drug delivery, tumor targeting, and immunomodulation. Their biocompatibility and ability to respond to environmental cues allow for precise and localized release of chemotherapeutic or immunotherapeutic agents directly within the tumor microenvironment [15]. This approach enhances treatment efficacy, minimizes systemic side effects, and can be further optimized by incorporating bioactive natural components that promote tissue regeneration and immune response modulation [16].

While natural hydrogels offer compelling advantages, they often face limitations in mechanical stability, long-term durability, and batch-to-batch reproducibility. To address these issues, synthetic hydrogels, such as those derived from poly(ethylene glycol) [17,18], poly(vinyl alcohol) (PVA) [19], or poly(acrylamide) (PAAm) [20], are sometimes employed due to their structural precision, tunable properties, and mechanical robustness. However, synthetic systems typically lack intrinsic bioactivity and may require chemical modification or blending with natural polymers to improve cell recognition and biocompatibility. In this context, hybrid hydrogels, combinations of natural and synthetic polymers, have emerged as a versatile solution, integrating the biological advantages of natural materials with the mechanical strength and tunability of synthetic counterparts [21]. Examples such as carboxymethyl cellulose (CMC)-PEG and alginate-PVA hybrids exhibit enhanced structural stability, controlled degradation, and improved drug encapsulation efficiency, while retaining cytocompatibility and biofunctionality [22,23].

Although synthetic and hybrid systems help overcome some practical limitations, it is the natural-origin materials that continue to inspire the design of next-generation bioactive hydrogels, capable of mimicking natural tissues and enabling advanced therapeutic applications in drug delivery, tissue engineering, and cancer treatment.

**Figure 1:**
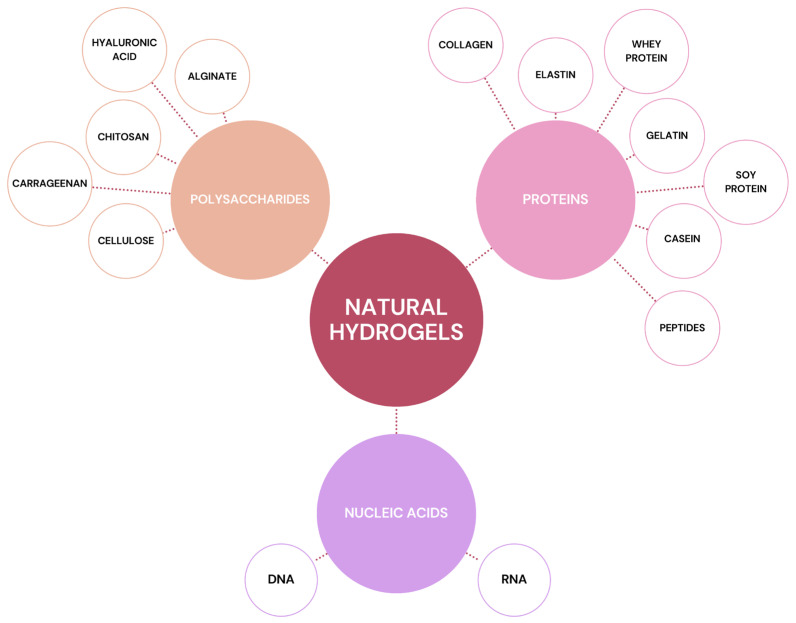
The three major categories of natural hydrogels: polysaccharide hydrogels (e.g., alginate, chitosan, hyaluronic acid), protein-based hydrogels (e.g., collagen, gelatin, fibrin), and nucleic acid-based hydrogels (e.g., DNA, RNA).

Naturally sourced biopolymers can be classified into three main categories based on their structural composition and functional properties (Figure 1). The first group comprises polysaccharides, including chitosan, hyaluronic acid, and alginate, which are widely used for their biocompatibility, biodegradability, and gel-forming capabilities [24]. The second group consists of protein-based biopolymers, such as gelatin, collagen, silk, and peptide-based systems, which offer excellent mechanical strength and bioactivity, making them suitable for biomedical and pharmaceutical applications [25]. Lastly, nucleic acids, such as DNA-based hydrogels, represent an emerging class of biopolymers with unique self-assembling properties and potential applications in gene delivery, tissue engineering, and biosensing [26].

## 4. Protein-Based Hydrogels

Among the commonly utilized natural biopolymers for hydrogel formation, proteins like gelatin, collagen, and silk stand out due to their excellent gelation properties. Gelatin, derived from the partial hydrolysis of collagen, is rich in cell-binding RGD sequences, which enhance bioactivity and cell adhesion [27]. Collagen, as a major ECM component, provides structural integrity and biological cues crucial for cell interaction and tissue support [28]. SF offers tunable gelation kinetics, mechanical strength, and biocompatibility, though it may lack intrinsic cell adhesion motifs, which can be supplemented by combining with gelatin [29]. These proteins can form hydrogels through enzymatic, chemical, or physical crosslinking, enabling control over mechanical properties, swelling behavior, and biodegradability [30]. Such hydrogels support cell growth and proliferation due to their biomimetic environment and suitable mechanical characteristics, and they also enable encapsulation of sensitive biomolecules. Advances in crosslinking techniques have helped overcome challenges like thermal instability and rapid degradation in physiological conditions, facilitating the development of stable and functional protein-based hydrogels [31].

Building on these advances, various natural proteins have been explored as structural components for hydrogel formation, each exhibiting distinct gelation mechanisms and functional properties. For example, Wang et al. used soy protein isolate [32] to prepare an emulsion gel through a salt ion-induced method. The addition of Ca^2+^ (0–7.5 mM) significantly increased the gel’s elasticity, creating a stiffer structure with better water-holding capacity. Interestingly, when the calcium concentration reached ten millimolar, the gel actually became weaker, showing a loose and non-uniform structure [33]. The characteristics of zein-based hydrogels were explored by Gagliardi et al., who found that zein could form stable dispersion gels at concentrations of 15% and 20% (*w*/*v*). Furthermore, high zein content promoted pseudoplastic behavior, offering a promising low-cost formulation for food production [34]. De Kruif et al. developed a transparent hydrogel using sodium caseinate (15%, *w*/*w*) and a transglutaminase (TGase) cross-linker at pH 5.7. The resulting casein hydrogels showed good water-holding capacity [35]. Beyond these systems, other proteins such as collagen, β-lactoglobulin, α-lactalbumin, bovine serum albumin (BSA), pea protein, and lactoferrin have also been employed to develop single-protein hydrogels. These materials exhibit diverse structural and functional properties depending on their amino acid composition and molecular conformation, allowing for fine-tuning of gel strength, porosity, and biodegradability [36].

A fundamental consideration in the design of protein-based hydrogels is the selection of the protein source, which dictates the material’s bioactivity, cost, and regulatory path. While natural proteins are broadly classified into animal-derived and plant-derived categories, each presents distinct trade-offs regarding clinical translation. Animal proteins, such as collagen and gelatin, offer superior biomimetic properties and inherent cell-signaling motifs (e.g., RGD sequences), yet they often suffer from significant batch-to-batch variability and potential zoonotic risks [37,38]. Conversely, plant-derived proteins like soy and zein provide a more sustainable and cost-effective alternative with enhanced reproducibility and a lower immunogenic profile [39,40]. However, plant sources typically lack the intrinsic biological “cues” found in the animal extracellular matrix, often necessitating chemical functionalization or hybridization with synthetic polymers to achieve the mechanical robustness and cellular recognition required for advanced therapeutic applications [21,41,42]. The primary trade-offs between these biological sources, specifically regarding their clinical translatability, reproducibility, and inherent bioactivity, are compared and summarized in Table 1.

While research on interactions among protein molecules has been extensive, binary protein hydrogels are a relatively emerging area of study. McCann et al. investigated the structural and rheological characteristics of soy protein-whey protein binary hydrogels. Upon heating to 95 °C, whey protein formed the primary network structure in composite gels, and when the concentration of whey protein decreased, the gel strength weakened. Soy protein [32] acted as a filler, and hydrophobic interactions between soy and whey proteins enhanced the gels’ elastic properties [43]. Additionally, Pan and Zhong developed stable nanohydrogels through co-assembly of zein and casein. After mixing zein with sodium caseinate at pH 11.5, the pH was adjusted to 7.0 to achieve co-assembly, resulting in spherical hydrogel nanoparticles smaller than 100 nm in diameter. These nanohydrogels remained stable for 30 days at 4 °C, and the freeze-dried nanoparticles showed excellent redispersibility [44]. Sarbon et al. [45] studied the microstructural, thermal, and rheological properties of gelatin-whey protein binary gels. When combined with gelatin (3%, 5%, and 10%), the composite hydrogels exhibited significantly higher elastic moduli compared to a 10% whey protein gel alone. This could be due to synergistic intermolecular interactions between the two proteins. The gel strength, structural integrity, and thermal stability also increased with higher gelatin concentrations. At lower concentrations, gelatin filled the network space of whey protein gels, whereas at higher concentrations, it induced the formation of a continuous gel network [46].

Another significant advantage lies in the structural and functional diversity of proteins, which enables precise drug targeting. Their inherent binding sites allow drugs to interact specifically with target molecules, while various targeting ligands can be conjugated to protein-based nanocarriers to further enhance site-specific delivery. This multifunctionality makes protein nanoparticles highly effective platforms for controlled drug delivery and tissue engineering applications [47].

Despite the promising potential of hydrogel-based systems for delivery, several critical limitations hinder their clinical translation. Among the most pressing challenges are the heterogeneity in tissue integration, the propensity for cell detachment at the hydrogel interface, and the instability or premature degradation of incorporated proteins [48]. These issues can severely compromise the therapeutic efficacy and reproducibility of the delivery platform, limiting their applicability in regenerative medicine and other biomedical contexts. To effectively translate preclinical successes into meaningful clinical outcomes, future research must prioritize the development of more robust and tunable protein release systems. This includes engineering hydrogels with spatiotemporally controlled release profiles that can be tailored to specific biological cues and tissue microenvironments. Moreover, an in-depth understanding of protein conformation, molecular interactions within protein-based hydrogels, and the mechanisms governing release kinetics is critical. These insights are indispensable for optimizing bioactivity preservation and ensuring sustained delivery at therapeutically relevant concentrations [49]. Equally important is the evaluation of biocompatibility, not only in terms of cytotoxicity but also considering immune responses, degradation byproducts, and long-term tissue remodeling. By addressing these multidimensional challenges, next-generation protein-based hydrogels can be rationally designed to achieve predictable, safe, and effective in vivo outcomes [50].

## 5. Synthesis Methods

**Figure 2:**
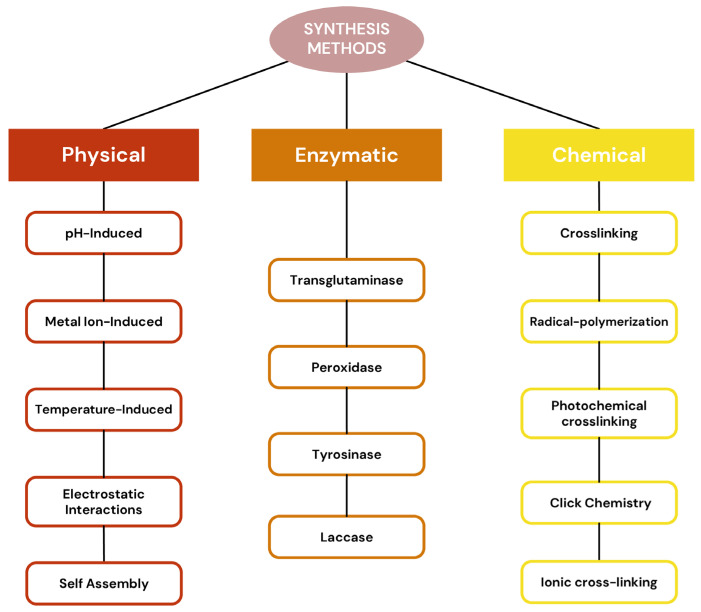
Schematic illustration of the key hydrogel synthesis pathways: physical, enzymatic and chemical crosslinking.

Protein-based hydrogels, recognized for their 3D network structures, can be synthesized through a wide range of strategies that exploit both covalent and noncovalent interactions among protein monomers and supramolecular assemblies. Depending on the synthesis method, the resulting hydrogels can exhibit distinct structural, mechanical, and functional properties, making them highly tunable for specific biomedical applications. These techniques include physical cross-linking, chemical cross-linking, enzymatic processes and self-assembly methods [51,52]. These synthesis strategies allow for precise control over the physical, chemical, and mechanical properties of protein-based hydrogels (Figure 2). The following sections provide a detailed overview of these techniques, highlighting representative examples and their implications for drug delivery, tissue engineering, and other biomedical applications.

### 5.1. Physical Synthesis Methods

The formation of protein hydrogels through physical methods depends on weak molecular interactions, including electrostatic forces, hydrogen bonding, and hydrophobic interactions. These approaches avoid the use of chemical cross-linkers, preserving the biocompatibility and functionality of the protein polymers [53]. For instance, hydrogen bonding between carboxylic groups of proteins like gelatin or collagen and other polymers can create pH-responsive gels with tunable mechanical properties [54]. Sun et al. investigated how charge distribution and ionic complementarity between oppositely charged peptide residues drive the lateral association of β-sheet nanofibers, forming nanostructures such as fibrils, bundles, and nanosheets. These assemblies create physically crosslinked networks through electrostatic and ionic interactions, which govern the hydrogel’s viscoelasticity and transition from viscous to self-supporting states. By tuning peptide charge patterns, ionic strength, and multivalent counterions, the density and stability of physical crosslinks can be precisely controlled, enabling biocompatible, chemically crosslinker-free hydrogels for bioengineering and biomaterial applications [55]. These physical synthesis methods are particularly advantageous for biomedical applications, enabling the development of injectable, self-healing hydrogels suitable for drug delivery and tissue engineering [56].

#### 5.1.1. pH-Induced Methods

Beyond electrostatic interactions, pH can also induce physical crosslinking that contributes to the formation and stabilization of hydrogel networks. By carefully adjusting the pH, protein solubility, charge distribution, and conformational state can be modulated, thereby promoting controlled aggregation and hydrogel formation. For example, at acidic pH, specifically around pH 3.5, BSA adopts an F-type (partially expanded cigarlike) conformation that facilitates aggregation and gelation. This conformational change is driven by electrostatic repulsions, which cause partial denaturation and expose hydrophobic core regions that are critical for protein aggregation despite remaining electrostatic repulsions. The gelation of BSA in this F isoform can occur at room temperature within 24 h or faster at 37 °C [57]. Conversely, under alkaline conditions, BSA experiences different conformational rearrangements that facilitate the formation of fluorescent hydrogels. These structures exhibit self-healing and multifunctional properties, making them suitable for biosensing and imaging applications [58]. This pH-driven approach is not limited to BSA. Similar strategies have been successfully applied to other protein systems. For instance, Tropoelastin, the soluble precursor of elastin and a key structural protein in the human body, adopts a distinctive α-helical, polyproline type II-like conformation when exposed to alkaline conditions with pH values above 10. This structural transition is driven by changes in electrostatic interactions and hydrogen bonding patterns, which promote the alignment of hydrophobic domains and facilitate subsequent self-assembly into elastin-like fibrillar networks. Such pH-induced conformational rearrangements are critical for controlling the material properties of elastin-based hydrogels, including their elasticity, stability, and capacity for biomimetic applications [59].

#### 5.1.2. Metal Ion-Induced Synthesis

Metal ions act as cross-linkers in protein hydrogel formation. Certain recombinant proteins interact with metal ions under light exposure, enabling the development of injectable, photoresponsive hydrogels [60]. Metal ions were able to induce crosslinking of whey protein nanofibrils, converting solutions into hydrogels. Higher-charged ions (Al^3+^, Sn^4+^) formed gels at lower concentrations, while monovalent ions (Na^+^, K^+^) needed higher concentrations. Additionally, multivalent ions such as Fe^3+^ or Co^2+^ can enhance the mechanical strength of hydrogels, supporting applications in biomedicine [61]. Whey proteins, particularly β-lactoglobulin, can form stable hydrogels upon cold gelation when pre-denatured and exposed to divalent metal ions such as Fe^2+^. The metal ions act as crosslinkers, promoting aggregation and network formation, with gel structure and stability strongly dependent on ion concentration and the pre-existing aggregation state of the proteins. Moreover, application of moderate electric fields during thermal denaturation modulates protein aggregation, enabling finer control over hydrogel microstructure and mechanical properties, thereby enhancing functional and nutritional performance [62].

#### 5.1.3. Temperature-Induced Synthesis

Thermal processing is another effective method for hydrogel formation. For instance, Whey protein-based hydrogels, when combined with lotus root amylopectin and heated at 95 °C, show enhanced mechanical stability, due to interactions and structural changes induced by heating that improve gel formation [63]. Cold-set hydrogels differ in that they are formed at low temperatures. This method is widely used for controlled drug release applications because it allows the encapsulation of temperature-sensitive bioactive compounds without degradation. For example, cold-set gels induced by additives like glucono-δ-lactone and sodium chloride are used to embed and release drugs in a controlled manner [64]. Protein aggregates formed by thermal treatment, such as amyloid fibrils and strands, can be further manipulated at room temperature by changing pH or salt concentrations to create cold-set gels. These cold-set gels often have superior mechanical properties, higher water absorption, and require lower concentrations for gelation compared to heat-set gels. Such gels are particularly useful for immobilizing charged bioactives for targeted and controlled release [65].

### 5.2. Chemical Synthesis Methods

Chemical cross-linking strategies establish strong covalent bonds between protein molecules, yielding hydrogels with improved stability. Chemical crosslinkers are classified as non-zero-length, which bridge adjacent polypeptides (e.g., glutaraldehyde (GLU), polyepoxides), and zero-length, which form direct covalent bonds between nearby reactive groups (e.g., 1-ethyl-3-(3-dimethylaminopropyl)-carbodiimide (EDC)) [66]. Wang et al. produced whey protein isolate fibrils via the thermal-acid method and subsequently crosslinked using citric acid (CA) to form cold-set hydrogels with tunable viscoelasticity. CA acted as a chemical crosslinker, inducing a phase transition from solution or soft-gel to rigid-gel states depending on pH and concentration. Optimal gelation occurred at 400 mmol/L CA under acidic conditions (pH 2) and at 100 mmol/L CA under neutral and alkaline conditions (pH 7–10). The enhanced storage modulus (G′) upon CA addition demonstrated its key role in promoting intermolecular crosslinking of whey protein isolate fibrils through pH-dependent interactions and charge modulation, resulting in stable, self-supporting hydrogels [67]. In another study, gelatin hydrogels were chemically crosslinked using GLU and glyceraldehyde (GAL) to study how cross-linker type, concentration, and solvent composition affect network formation and stability. GLU efficiently produced stable, highly crosslinked hydrogels under all tested conditions, whereas GAL required higher concentrations and acetone levels, likely due to acetone’s dehydrating effect, enhancing GAL reactivity through Schiff base formation with gelatin’s amine groups [68].

### 5.3. Enzyme-Induced Synthesis

Enzymatic cross-linking is a bioinspired method for forming protein-based hydrogels that leverages enzymes to catalyze the formation of covalent bonds between protein molecules. This approach offers several advantages over traditional chemical crosslinking, such as mild reaction conditions and enhanced biocompatibility [69]. Moreover, enzymes can target specific amino acid residues in proteins, allowing more controlled and site-specific crosslinking, therefore improving mechanical properties and facilitating functionalization. Common enzymes used include TGase, tyrosinase, and horseradish peroxidase (HRP), which catalyze cross-linking through different amino acid side chains [70]. Sahoo et al. provided a comprehensive investigation into how HRP-mediated crosslinking can be harnessed to modulate and enhance the mechanical properties of SF hydrogels through manipulation of silk extraction conditions. Their study offered direct evidence of dityrosine crosslinking catalyzed by HRP, and mechanical characterization revealed markedly increased stiffness, resilience, and β-sheet content in HRP-crosslinked silk hydrogels [71]. The study on an injectable phosphocreatine-grafted gelatin hydrogel incorporating bioactive particles underscores the potential of enzyme-catalyzed protein hydrogels as multifunctional scaffolds that facilitate synergistic tissue regeneration through precise biochemical and structural modulation. TGase-mediated crosslinking enhanced the structural and functional properties of the scaffold, facilitating mild and efficient covalent bonding and enabling the incorporation of hierarchically structured teriparatide/strontium–zinc phosphate-functionalized Zn–Cu particles without compromising bioactivity [72]. These works exemplify the advantages of enzyme-mediated crosslinking as a biologically compatible strategy for developing advanced protein hydrogels in biomedical applications.

The wide range of available synthesis methods provides the ability to finely tune the properties of protein-based hydrogels, making them highly adaptable and suitable for various applications across biomedicine, drug delivery, and biomaterials science.

## 6. Protein-Based Hydrogels for Drug Delivery

Protein-based hydrogels have emerged as highly versatile platforms for drug delivery, owing to their intrinsic biocompatibility, tunable biodegradation, and ability to form highly hydrated, 3D networks. Their natural origin provides inherent bioactivity and structural motifs that can interact favorably with therapeutic molecules, while their responsiveness to environmental cues, such as pH, temperature, or enzymatic activity, enables controlled and stimuli-responsive release profiles [73]. Moreover, the diverse chemical functionalities present in proteins allow for a broad range of crosslinking strategies, facilitating the design of hydrogels with tailored mechanical properties, stability, and drug-loading capacity.

**Figure 3:**
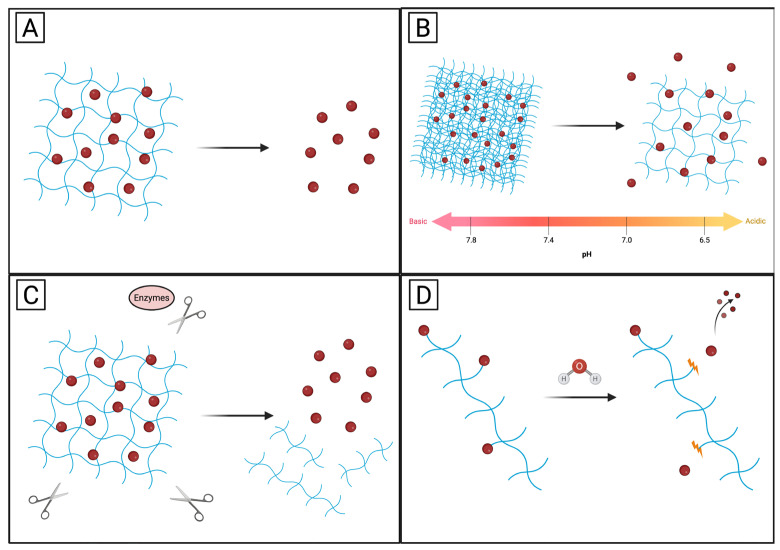
Mechanistic pathways of drug release in protein-based hydrogel systems. (**A**) Diffusion: Passive movement of therapeutic cargo through the aqueous pores of the scaffold. (**B**) Swelling: Responsive expansion of the network triggered by external triggers (pH/temperature), increasing pore volume. (**C**) Erosion: Controlled biodegradation of the protein backbone by endogenous enzymes (e.g., matrix metalloproteinases) in the tumor microenvironment. (**D**) Dissociation: Cleavage of covalent or electrostatic interactions between the drug and the protein chain.

As illustrated in Figure 3, protein-based hydrogels offer highly versatile and precisely controllable platforms for therapeutic delivery by integrating multiple release mechanisms within a single material system. The most fundamental mode of release occurs through passive diffusion (Figure 3A), in which drug molecules migrate from the hydrogel matrix into the surrounding tissue along a concentration gradient. This mechanism is primarily governed by the mesh size of the protein network, the molecular weight of the therapeutic agent, and the degree of hydrogel crosslinking [74]. Beyond simple diffusion, “smart” protein hydrogels are frequently engineered to respond dynamically to pathological cues. Stimuli-responsive swelling (Figure 3B) enables the hydrogel to undergo reversible volumetric changes in response to environmental conditions such as pH. In the context of cancer therapy, exposure to the acidic tumor microenvironment can induce hydrogel expansion, increasing mesh permeability and thereby accelerating drug release in a spatially selective manner [75]. Protein-based hydrogels also possess intrinsic biodegradability due to their peptide backbone, which can be exploited for enzyme-triggered release (Figure 3C). Tumor-associated proteases, often overexpressed by malignant cells, can selectively cleave peptide sequences within the hydrogel scaffold. This enzymatic erosion gradually disrupts the matrix architecture, enabling sustained and localized release of the encapsulated therapeutic payload while minimizing off-target exposure [76]. In addition to physical entrapment, therapeutics may be chemically conjugated to the protein network through cleavable linkages (Figure 3D). These drug–protein conjugates are designed to remain stable during circulation but dissociate upon exposure to specific chemical, enzymatic, or redox triggers present in diseased tissues. This strategy provides an additional layer of control, ensuring that drug activation and release occur only under well-defined biological conditions, thereby enhancing therapeutic efficacy and reducing systemic toxicity [77].

Beyond their tunable synthesis and release properties, protein-based hydrogels play a critical role in protecting encapsulated therapeutics during transit through harsh physiological environments, particularly in oral delivery, where labile biologics are otherwise rapidly degraded by gastric conditions [78]. This protective function is especially relevant for oral and mucosal delivery routes, where drugs are exposed to extreme pH conditions, digestive enzymes, and mechanical stress prior to reaching their site of action [48]. A key mechanism underlying this protection is the pH-dependent conformational behavior of protein networks [79]. Upon exposure to acidic environments, such as the gastric compartment (pH 1.2–2.0), which is often close to the isoelectric point (pI) of many proteins, the hydrogel undergoes pronounced structural collapse [80]. At the pI, electrostatic repulsion between protein chains is minimized, leading to network densification and a significant reduction in mesh size. This conformational collapse generates substantial steric hindrance, effectively forming a physical barrier that limits the diffusion and penetration of proteolytic enzymes, including pepsin, thereby shielding the encapsulated therapeutic cargo from premature degradation [81]. In parallel, protein-based hydrogels exhibit intrinsic buffering capacity due to the high density of ionizable amino acid side chains within their structure [82]. These functional groups partially neutralize the surrounding acidic medium, creating a localized microenvironment within the hydrogel core that is less acidic than the bulk gastric fluid. This buffering effect further contributes to preserving the structural integrity and bioactivity of pH-sensitive compounds, including fragile peptides, proteins, and hydrophobic small molecules [83]. Importantly, this protective shielding is reversible: upon transition to environments with near-neutral pH, such as the small intestine, electrostatic repulsion between protein chains is restored, promoting hydrogel re-expansion and increased network permeability. This pH-triggered swelling enables controlled and site-specific release of the therapeutic payload, either through diffusion, enzymatic erosion, or cleavage of labile drug–protein linkages [84]. Collectively, these mechanisms highlight how protein-based hydrogels function not only as delivery matrices but also as active protective carriers, ensuring drug stability during transit and enhancing therapeutic efficacy.

Table 2 summarizes commonly used protein sources for hydrogel fabrication, highlighting their main features, crosslinking approaches, and therapeutic relevance in drug delivery. In the next section, we will discuss the characteristics of several representative proteins and present examples of their application as drug delivery systems, with particular emphasis on strategies developed for cancer treatment.

### 6.1. Gelatin

Gelatin is a denatured protein obtained through the acidic hydrolysis of animal collagen. This biomolecule has been utilized for many years across the pharmaceutical, cosmetic, and food industries. One of the notable effects of gelatin is its ability to stimulate the immune system due to its denaturation process. Gelatin functions as a polyampholyte, possessing both cationic and anionic active groups, as well as hydrophobic groups, in a balanced ratio of 1:1:1. This results in a gelatin molecule with approximately 13% positive charge (from amino acids like lysine and arginine), 12% negative charge (from glutamic and aspartic acids), and 11% hydrophobic amino acids (including leucine, isoleucine, methionine, and valine) [85]. The remaining structure is primarily composed of glycine, proline, and hydroxyproline. Cationic gelatin is derived from type 1 pig skin collagen through acidic hydrolysis, while anionic gelatin is obtained from bovine collagen via hydrolysis. Gelatin is employed in various drug formulations for systemic use, serving clinically as a plasma volume expander and a stabilizer in protein formulations, vaccines, and gelatin-based sponges like gel foam [86]. The sequence of arginine-lysine-glycine is crucial in many ECM proteins, facilitating cell binding and communication by interacting with the beta subunit of integrin receptors on cell surfaces. This property gives gelatin a significant advantage over synthetic polymers that often lack specific cell recognition and binding sites. Furthermore, the active groups within gelatin allow for various chemical modifications, either directly or through different linkers, which is particularly valuable for developing targeted drug delivery systems and for attaching substantial amounts of drugs to the carriers [47]. Among its various forms, gelatin hydrogel serves as an effective sustained-release carrier due to its remarkable water retention and viscoelastic properties. It can absorb water and re-swells after being dehydrated under specific pressure conditions [87]. Current research indicates that gelatin hydrogels can be synthesized through various methods, including chemical crosslinking [88], temperature-induced crosslinking [89], photo-crosslinking [90], and enzyme-mediated crosslinking [91]. However, the properties of hydrogels produced via temperature and photo-crosslinking are often suboptimal due to varying environmental conditions [92].

#### Drug Delivery Applications and Cancer Treatment

Owing to their intrinsic biodegradability and capacity to form stable 3D networks, gelatin-based hydrogels represent a promising class of materials for advanced drug delivery systems [93]. Recently, a phenylboronic-acid-modified gelatin methacryloyl hydrogel was developed to release epigallocatechin gallate (EGCG) in response to high reactive oxygen species (ROS) and low pH, reducing inflammation in intervertebral disk degeneration models. Phenylboronic acid was used to introduce functional groups enabling UV crosslinking and dynamic boronic ester formation. The resulting hydrogel exhibited high swelling capacity, tunable mechanical strength, and enzymatic degradability, all essential for biomedical applications, and protected nucleus pulposus cells while maintaining disk structure in vivo [94]. Besides serving as an excellent carrier for bioactive molecules, gelatin also exhibits intrinsic biological activity that promotes tissue regeneration and healing [6]. Canafístula et al. developed an innovative hydrogel system by combining gelatin with the carbohydrate-based polymer guar gum, harnessing gelatin’s natural biocompatibility and hydrophilicity alongside the mechanical robustness and chemical reactivity of oxidized guar gum crosslinked via Schiff base chemistry. The resulting hydrogels displayed tunable physicochemical properties dependent on gelatin content, including variations in gelation rate, crosslinking density, porosity, and degradation behavior. Notably, the gelatin–guar gum hydrogels exhibited strong intrinsic antibacterial activity against methicillin-resistant *Staphylococcus aureus* (MRSA) and other staphylococcal strains, even in the absence of added antibiotics. Moreover, they demonstrated excellent cytocompatibility, non-irritant behavior, and desirable functional attributes such as adhesiveness, injectability, and self-healing, underscoring their potential for topical drug delivery applications, particularly in wound healing, dermatitis, and ophthalmic treatments [95]. In another work, a dopamine- and [2-(methacryloyloxy) ethyl] dimethyl-(3-sulfopropyl) ammonium hydroxide (SBMA)-modified gelatin hydrogels were developed via an in situ dopamine-triggered reaction in the presence of zinc sulfate (ZnSO_4_). This dual organic-inorganic network imparted biomimetic adhesion, self-healing ability, and thermal resilience. The zinc ions served both as crosslinkers and as bioactive agents, facilitating sustained antibacterial action. The gelatin matrix provided a biocompatible, hydrophilic, and porous substrate suitable for controlled release of therapeutic molecules or ions. The modified gelatin hydrogels exhibited complete antibacterial activity (up to 100%) against *E. coli* and *S. aureus*, demonstrating that gelatin’s intrinsic biopolymeric structure can be engineered for combined drug or ion release and surface antimicrobial defense [96].

Gelatin-based hydrogels have also been extensively explored as delivery platforms for anticancer drugs. Their biocompatibility, biodegradability, and ability to form stable yet stimuli-responsive networks enable controlled and localized drug release at tumor sites, minimizing systemic toxicity. Moreover, the presence of functional groups within the gelatin backbone allows chemical modification and conjugation with targeting ligands, nanoparticles, or chemotherapeutic agents, further enhancing therapeutic efficacy and specificity in cancer treatment [97]. An et al., for example, effectively encapsulated doxorubicin (DOX) and rifampicin in a dual-reinforced gelatin methacrylate (GelMA) hydrogel modified with N′-(2-nitrobenzyl)-N-acryloyl glycinamide (NBNAGA), forming a UV-crosslinked photoresponsive network. The structure combines chemical and physical crosslinking, enhancing mechanical strength and stability. Hydrophobic drugs were released in a temperature- and light-dependent manner due to the photo-cleavable nitrobenzyl groups. The hydrogels demonstrated controlled drug release and strong antibacterial activity against *E. coli* and *S. aureus*, making them highly effective for wound-healing drug delivery applications. In vivo tests in mice confirmed accelerated skin regeneration, highlighting their utility as biocompatible wound dressings with on-demand hydrophobic drug release [98]. An injectable gelatin hydrogel composite integrating Pluronic-based micelles and gelatin microgels has been synthesized to co-deliver a hydrophobic drug (curcumin) and a hydrophilic drug (5-fluorouracil, 5-FU). The gelatin component contributes biocompatibility, injectability, and biodegradability, while serving as a hydrophilic matrix capable of controlled drug encapsulation and release. The Pluronic micelles formed hydrophobic domains for curcumin entrapment, whereas the microgels encapsulated 5-FU, enabling a dual-release mechanism tailored for synergistic chemotherapy. Drug release assays confirmed sustained 5-FU release exceeding one month and controlled curcumin diffusion from the micellar domains. The composite hydrogels exhibited significant antiproliferative effects against human colorectal adenocarcinoma HT-29 cells, with improved cell death and reduced proliferation compared to single-drug systems. Gelatin’s matrix integrity and hydrophilic nature facilitated both injectability and the maintenance of drug stability, making this composite system a powerful biomaterial for localized, combinational cancer therapy [99]. The study of Ullah et al. emphasizes gelatin’s capacity to form stable, pH-sensitive networks that can protect the encapsulated oxaliplatin (OXP) through the gastrointestinal tract and enable gentle, controlled release in the colonic environment. The presence of functional groups within gelatin allowed for chemical modifications with acrylic acid (AA) and 2-acrylamido-2-methylpropane sulfonic acid (AMPS) that were able to fine-tune the hydrogel’s swelling, degradation and drug release profiles. The OXP-loaded hydrogels exhibited dose-dependent cytotoxicity against Vero, MCF-7, and HCT116 cell lines and demonstrated excellent biocompatibility and oral tolerability in animal studies. This approach aimed to improve the targeted and sustained delivery of the anticancer drug, reducing systemic side effects and increasing therapeutic efficacy in colorectal cancer (CRC) treatment [100].

### 6.2. Silk

Silk is a natural fibrous protein mainly produced by silkworms (such as Bombyx mori) and spiders. It possesses a range of characteristics that make it an ideal material for biomedical applications. It is biocompatible and enzymatically biodegradable, ensuring safe integration into biological systems without inducing adverse reactions [101]. Additionally, silk-based materials exhibit low immunogenicity and are nontoxic, making them suitable for medical use [102]. One of silk’s key advantages is its exceptional mechanical stability, along with its controllable format and size. Its reversible swelling behavior allows for storage in a dried state, thereby expanding its potential for functionalization, fabrication, and processing into robust biomaterials. The unique physicochemical properties of silk stem from the structural organization of its proteins at the primary and secondary levels. The interplay between highly repetitive hydrophobic crystalline regions and hydrophilic non-crystalline domains results in a hierarchical structure that drives self-assembly. This structural organization leads to strong physical interactions and outstanding mechanical properties, including high strength and toughness [103]. Furthermore, the hydrophobic domains within silk proteins, specifically, the GAGAGS motif in silkworm silk and the poly(GA) and poly(A) sequences in spider silk, contribute to its crystalline content. These domains play a crucial role in enhancing hydrophobic interactions with drug molecules, thereby enabling precise control over drug loading and release kinetics. Advancements in genetic engineering have further expanded the versatility of silk-based biomaterials. By modifying synthetic silk genes, researchers can regulate the balance between crystalline and noncrystalline domains, thereby fine-tuning both the mechanical properties of silk and its drug-binding capabilities. This ability to manipulate silk’s molecular structure opens new possibilities for optimizing its application in controlled drug delivery systems [103,104]. Silk consists primarily of two proteins: fibroin, which forms the structural core of the fiber, and sericin, a glue-like coating that binds the fibroin filaments together. Fibroin features a distinctive molecular architecture enriched with repetitive amino acid sequences (chiefly glycine, alanine, and serine) that assemble into tightly packed antiparallel β-sheet crystals. This ordered structure imparts silk with its exceptional mechanical strength, toughness, and biocompatibility [105]. Within SF, the protein chains adopt β-sheet secondary structures, providing rigidity and stability while retaining a degree of flexibility. In contrast, sericin is a hydrophilic protein rich in polar groups, serving both as an adhesive and a protective layer for the fibroin core [106]. SF’s excellent histocompatibility and low immunogenicity make it unlikely to provoke significant immune responses, and it possesses a degree of biodegradability, typically breaking down into amino acids or oligopeptides. The degradation products are not harmful to the body and can provide nutritional and reparative benefits to surrounding tissues, leading to its widespread application in tissue engineering [17]. SF hydrogels can be synthesized using both physical and chemical crosslinking methods. Physical crosslinking leverages the silk protein’s sensitivity to various molecular conditions (such as pH, shear, and vibration) to induce the formation of beta-folded structures that yield hydrogels. Common physical crosslinking techniques involve elevated temperatures and adjustable pH levels, often utilizing cyclone processing or ultrasonic processing [107]. While SF hydrogels produced through physical crosslinking exhibit considerable strength, they have drawbacks, including prolonged crosslinking times and brittleness. Chemical crosslinking for SF hydrogels involves creating covalent bonds between polymer chains [108]. Commonly employed methods include the use of crosslinking agents, light exposure, and enzymatic reactions [109]. Sericin, another silk-derived protein, encases SF and offers protective benefits. The primary distinction between the two is that SF swells in water but does not dissolve, while sericin can dissolve in hot water [110]. Research into sericin has unveiled its considerable advantages as a biomaterial, including its stable source, strong processability, hydrophilicity, low immunogenicity, ability to promote cell proliferation, inhibition of tyrosinase activity, and controllable degradation. In the context of sustained-release drug delivery systems, sericin can be engineered into hydrogels using a range of strategies, including physical, chemical, and photo-crosslinking methods. The most common chemical approach involves GLU as a crosslinking agent [111]. Alternatively, based on the principle that proteins are insoluble in ethanol, pure sericin hydrogels can be fabricated through ethanol-induced precipitation combined with ultrasonic treatment [112]. Moreover, photo-crosslinking has also been explored: for example, Qi et al. developed an in situ hydrogel by UV-induced photo-crosslinking of methylacryloyl-functionalized sericin, demonstrating a controllable and biocompatible approach to hydrogel formation [113]. Gels derived from this protein exhibit sensitivity to pH variations, making them suitable for developing smart drug delivery systems.

#### Drug Delivery Applications and Cancer Treatment

**Figure 4:**
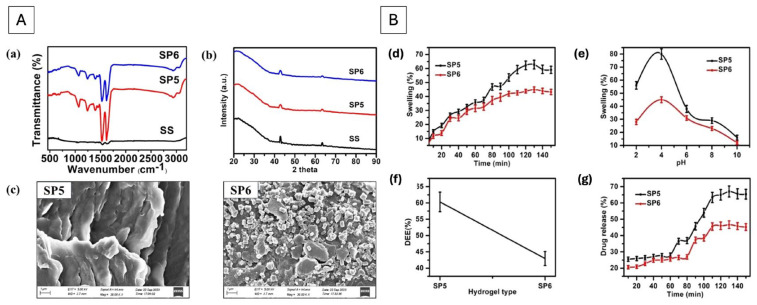
(**A**) Structural characterization of silk-based hydrogels: (**a**) FTIR spectra; (**b**) XRD pattern; (**c**) SEM images of the hydrogel samples; (**B**) Drug release kinetics of silk-based hydrogels: (**d**) Zero-order kinetic model; (**e**) Pseudo-first-order kinetic model; (**f**) Korsmeyer–Peppas kinetic model; and (**g**) Higuchi kinetic model [114]. Reproduced with permission from “Copyright © 2024 The Authors. Published by American Chemical Society”.

Silk-based hydrogels have emerged as versatile and biocompatible platforms for drug delivery, offering tunable mechanical properties, controlled degradation, and the ability to sustain the release of therapeutic agents over prolonged periods. Recently, a soft hydrogel was formulated at room temperature by blending sericin and polycaprolactone (PCL), achieving a porous and non-Newtonian rheological system with good absorption capacity. The internal morphology of such silk-based systems, as characterized by scanning electron microscopy (Figure 4A), typically reveals an interconnected porous architecture. This porosity is critical not only for drug loading but also for providing the gas exchange necessary for tissue regeneration. In these blends, sericin’s inherent properties contributed importantly to the hydrogel’s antibacterial efficacy against both Gram-positive and Gram-negative bacteria, further enhanced when loaded with the model drug diclofenac sodium. From a mechanistic standpoint, these hydrogels demonstrated substantial swelling and sustained controlled release behavior. To quantitatively evaluate these profiles, experimental data are often fitted to mathematical models as shown in Figure 4B. For silk-PCL systems, the release kinetics typically aligned with the Korsmeyer–Peppas model, where the release exponent often indicates an anomalous transport mechanism, a combination of Fickian diffusion through the sericin-rich pores and the slow erosion of the PCL-stabilized matrix. This sustained release, coupled with the natural bioactivity of silk, makes these platforms particularly promising for chronic wound healing and long-term localized drug delivery application [114].

Ghorbani et al. combined alginate and SF, crosslinked through both ionic gelation and Schiff-base reactions, resulting in a scaffold with enhanced mechanical strength, stability, and biocompatibility. Although primarily developed for bone tissue engineering, the system’s properties (porosity, sustained degradation, and tunable crosslinking density) also make it suitable for localized drug delivery, including anticancer therapies at bone defect sites. SF contributed by forming β-sheet domains that stabilized the network and modulated drug binding, enabling controlled, site-specific release of therapeutic molecules [115]. Peng et al. developed an SF-based hydrogel that can self-assemble into injectable, porous networks under physiological conditions. Their study emphasizes the biocompatibility, biodegradability, and excellent mechanical properties of the protein, making it suitable for minimally invasive delivery. The hydrogel incorporates iodine as a therapeutic agent, which, when released in a sustained manner, induces apoptosis in osteosarcoma cells through mechanisms involving ROS and apoptosis pathways. The hydrogel’s X-ray visibility allowed for image-guided injection directly into tumor sites, facilitating precision treatment. SF formed a stable, biocompatible, and imageable hydrogel, able to combine therapeutic and diagnostic functionalities in a single system, making it a promising platform for focal osteosarcoma therapy [116]. Besides bioactive molecules for tumor therapy, silk-based hydrogels have also been explored as delivery systems for conventional chemotherapeutic agents, offering both local administration methods, such as intratumoral and transdermal delivery, and systemic approaches via intravenous injection, and in various formats [117]. For localized drug delivery, 3D silk implants have demonstrated effective control over drug release rates, including injectable hydrogels designed for sustained drug administration [118,119]. For systemic delivery, the study of Fernández-Serra et al. describes SF hydrogels exhibiting notable permselectivity, enabling controlled diffusion of drug molecules and making them highly effective carriers for advanced neurotherapeutic delivery. Their tunable network structure allowed precise regulation of drug permeation, ensuring sustained and targeted release to neural tissues while minimizing systemic exposure. Such hydrogels protected encapsulated agents from premature degradation, maintained therapeutic concentration at disease sites, and supported localized administration of chemotherapy drugs for neuro-oncological applications. This selective barrier function enhanced efficacy and safety in complex neural environments [120,121]. Jaiswal et al. developed injectable hydrogels composed of a blend of Bombyx mori silk fibroin (BMSF) and Antheraea assamensis silk fibroin (AASF) to evaluate their potential for localized drug delivery in post-lumpectomy breast cancer treatment [122,123]. A 3D in vitro lumpectomy model using the MDA-MB-231 cell line was designed to assess the efficacy of these hydrogels in delivering DOX for the targeted elimination of residual breast cancer cells. Additionally, the potential for adipose tissue regeneration in the lumpectomy site was explored by incorporating dexamethasone (DEX) into the hydrogel system. Rheological analysis demonstrated that the BMSF/AASF blended hydrogels possessed viscoelastic properties and injectability suitable for minimally invasive applications. The slow and sustained release of DOX from the hydrogels resulted in effective cytotoxicity against MDA-MB-231 cells, as confirmed by in vitro studies. These findings highlight the potential of the developed injectable hydrogels as a dual-purpose system for localized anticancer drug delivery and post-lumpectomy breast reconstruction [122]. A. Gangrade and B.B. Mandal developed an innovative porous silk scaffold designed to integrate both a soft hydrogel matrix and stomach cancer (AGS) cells within a single platform. The AGS cells were seeded around the periphery of the scaffold, where they occupied the porous structure and formed 3D spheroids. Meanwhile, an injectable silk hydrogel embedded with cisplatin-loaded nanocomposites was introduced into the central cavity of the scaffold to assess its extended bioactivity over 11 days. This strategic arrangement allowed for sustained cisplatin release, ensuring prolonged exposure of the drug to the surrounding spheroids for enhanced therapeutic efficacy. To simulate cancer recurrence, AGS cells were reintroduced on the second day of treatment. Experimental results demonstrated that the nanocomposite silk hydrogel significantly prolonged the stability and cytotoxic effects of cisplatin. Consequently, the reseeded AGS cells were unable to survive on the scaffold, highlighting its potential to prevent tumor relapse [124]. These approaches underscore the effectiveness of the engineered silk scaffolds in supporting targeted and sustained chemotherapy while minimizing the likelihood of cancer recurrence.

### 6.3. Soy Protein

Soy protein [32] is a naturally derived polymer extensively explored as a foundational material for the development of polymeric networks designed for drug delivery applications. Its widespread availability, favorable water solubility, and exceptional biocompatibility, biodegradability, non-immunogenicity, and anti-carcinogenic properties make it a promising candidate for biomedical use [125]. Extracted from an abundant, cost-effective, and renewable plant-based resource, SP consists primarily of two globular protein subunits: 7S (β-conglycinin) and 11S (glycinin) [126]. Due to its inherent bioactivity, SP has found extensive applications in various fields, including the production of adhesives, hydrogels, plastics, films, coatings, and emulsifiers. Additionally, it is widely investigated for its potential in biotechnology and biomedical engineering [127]. Compared to other biodegradable polymers and natural proteins, the SP matrix exhibits distinctive advantages, such as improved water resistance, prolonged storage stability, and structural robustness. These characteristics position SP as a valuable material for the design of novel biomaterials and medical devices, further enhancing its potential in biotechnology and biomedical research [128]. SP isolate [32] is the enriched form of soy protein, offering a balanced mix of polar, non-polar, and essential amino acids, making it suitable for use in various pharmaceutical applications. In aqueous environments, SPI proteins assemble into spherical structures with a hydrophilic outer shell and hydrophobic core, facilitating stability and functionality. Upon the introduction of precipitating agents or crosslinkers, SPI can form diverse structures, including microspheres and hydrogels, expanding its potential as a carrier in drug delivery systems. The distinctive 11S/7S globulin ratio further influences the emulsifying, gelling, and foaming properties relevant for biomedical uses [129].

#### Drug Delivery Applications and Cancer Treatment

The integration of SP-based hybrid hydrogels into drug delivery applications has gained significant attention in recent years, owing to their renewable, biodegradable, and biocompatible nature. Additionally, their tissue-mimicking properties make them highly desirable in biomedical engineering [130]. Singhal et al. developed a pH-sensitive, biocompatible hydrogel by grafting 2-hydroxyethyl methacrylate (HEMA) onto SPI. This grafting approach imparted significant stimulus-responsive behavior to the SP hydrogel, making it adaptable for controlled drug delivery applications in biomedical contexts. SIP served as the core biopolymer matrix in these hydrogels, supporting excellent cell adhesion and growth. They used paracetamol as the model drug for loading and release experiments, with results showing effective encapsulation and sustained release and demonstrating its suitability for pH-sensitive, biomedical drug delivery applications [131]. Another study reports the development of SPI-based hydrogels for probiotic delivery. The hydrogels were formed through enzymatic crosslinking by microbial transglutaminase (mTGase), creating a biocompatible and stable network under mild conditions that preserves probiotic viability. The authors used ultrasonic treatment to enhance protein dispersion and gel uniformity, resulting in a denser and more elastic structure with improved mechanical strength. The addition of citrus pectin further stabilized the matrix and contributed to protection against acidic and UV stress during gastrointestinal transit, confirming the platform’s promising capacity for targeted oral delivery of probiotics and other sensitive bioactives [132]. A nanocomposite hydrogel combining SP and PAAm was synthesized via in situ polymerization. The SP served as a natural, biodegradable, and biocompatible matrix, while the PAAm provided mechanical strength and enhanced water absorption properties. Crucially, this hydrogel demonstrated sustained and controlled release of the antibiotic ciprofloxacin, with drug release rates reaching up to 95% in vitro. The network structure of the hydrogel, characterized by good porosity and layered morphology, supported effective drug loading and gradual release. This study highlights the potential of SP-based nanocomposite hydrogels as versatile drug delivery systems, combining natural protein properties with synthetic polymer advantages for controlled antimicrobial therapy. The hydrogel’s pH-responsive swelling behavior further enables targeted release applications [133].

Building upon the general advances in drug delivery, particular attention has been devoted to the use of SP–based hydrogels for the targeted and controlled delivery of both hydrophobic and hydrophilic chemotherapeutic agents, aiming to enhance antitumor efficacy while minimizing systemic toxicity. Their natural gelation properties and capacity for modification allow for controlled drug release profiles, improving drug stability and targeting precision in cancer therapy [134]. Viale et al. developed the first SPI-based hydrogel incorporating DOX-loaded liposomes (LIPO-DOX) for the localized treatment of recurrent glioblastoma. The implantable, in situ-gelling hydrogel was designed to conform to the post-surgical cavity, enabling sustained drug release while being biodegradable, thus avoiding the need for surgical removal. Complete degradation was estimated to occur within two months. The encapsulation efficiency of LIPO-DOX was high (~94%), and the liposomes exhibited a characteristic “coffee bean” morphology within the protein matrix. Release studies showed gradual liposome diffusion over 48 h, and in vitro assays on U87-MG glioblastoma cells confirmed that released LIPO-DOX maintained pharmacological activity, effectively reducing cell viability in a dose-dependent manner [135].

### 6.4. Casein

Caseins represent the predominant protein group in milk, comprising approximately 80% of its total protein content. These proteins are encoded by multiple genes clustered on the same chromosome [136]. The primary casein types include αS1-casein (Bos d 9), αS2-casein (Bos d 10), β-casein (Bos d 11), and κ-casein (Bos d 12), contributing approximately 40%, 12.5%, 35%, and 12.5% of the total casein fraction, respectively. Due to genetic polymorphisms, caseins exhibit high structural variability, resulting in multiple protein variants. These variations arise from single amino acid mutations, deletions of peptide segments of varying lengths, or post-translational modifications such as glycosylation, phosphorylation, and partial hydrolysis, all of which influence their physicochemical properties and allergenic potential [137]. As phosphoproteins, caseins contain between one and eleven phosphate groups, which form ester bonds primarily with serine hydroxyl residues. The number of phosphorylated serine residues within the polypeptide chains determines their affinity for calcium ions [138]. Functionally, αS1-, αS2-, and β-caseins serve as calcium-binding proteins, whereas κ-casein plays a crucial role in stabilizing the casein micellar structure [139]. Notably, casein demonstrates remarkable ion-binding capacity, surface activity, self-assembly behavior, emulsification abilities, and gel-forming properties, all of which contribute to its potential as a controlled drug release system [47]. Gelation of casein typically involves pH-triggered mechanisms where changes in pH alter protein charge and promote aggregation and network formation. Enzymatic crosslinking using TGase has been demonstrated to enhance the gel network by increasing fractal dimension and creating a tighter, more stable matrix [140]. These hydrogels show advantageous properties such as mechanical strength, stability, biocompatibility, and controlled drug release behavior. Functionalization with other polymers like konjac glucomannan or polyglutamic acid (PGA) further modulates these properties, enabling tailored biomedical applications, including drug delivery and tissue engineering [141,142].

#### Drug Delivery Applications and Cancer Treatment

As the primary protein component of milk, casein has gained increasing recognition for its versatility in drug delivery systems. Its key advantages include cost-effectiveness, stability, and the ability to encapsulate a diverse range of bioactive molecules. Due to its distinctive physicochemical characteristics, casein has been extensively studied for controlled drug release. It possesses a strong affinity for binding various ions and bioactive compounds, exhibits outstanding emulsification and gelation capabilities, and retains water effectively, making it a valuable component for pharmaceutical formulations [143]. Despite its advantages, casein-based drug delivery systems are not without challenges. Potential immunogenic concerns, including allergenic responses and immunosuppressive effects, must be taken into account. While casein undergoes enzymatic breakdown into amino acids in the gastrointestinal tract, intact casein proteins may still trigger immune responses, particularly in intravenous applications [144]. Wang e t al. explored the enzymatic crosslinking of casein with γ-polyglutamic acid (γ-PGA) to form hydrogels using mTGase and they tested both hydrophilic vitamin B12 and hydrophobic aspirin as model drugs. Casein served as a natural protein matrix with abundant reactive glutamine and lysine residues that were targeted by mTGase to create covalent bonds, resulting in a stable, biocompatible hydrogel network. This enzymatic gelation method provided mild reaction conditions and precise control over hydrogel properties, making it highly suitable for encapsulating and delivering various bioactive drugs. The resulting casein-γ-PGA hydrogels exhibited controlled drug release profiles and enhanced mechanical stability, positioning them as promising candidates for sustained and targeted drug delivery applications [145]. Casein has been combined with other natural proteins, such as gelatin, to enhance the mechanical strength, stability, and functional properties of the resulting hydrogels. Zhang et al., for example, developed a biocompatible and biodegradable hydrogel composed of gelatin and casein proteins, enzymatically crosslinked to form a natural hydrogel network for wound dressing applications. An innovative feature of this hydrogel system is its on-demand tissue adhesion capability, mediated by the application of chitosan solution, which enhances the hydrogel’s adhesion strength and allows easy control and activation at the wound site. The hydrogel’s degradation rate and drug release profile could be finely tuned by adjusting enzymatic crosslinking conditions. For drug delivery, the study used tetracycline hydrochloride as a model drug loaded into the hydrogel matrix. The casein-containing hydrogel provided effective antibiotic delivery directly at the wound site, offering antibacterial properties essential for preventing infection in wound healing. The drug-loaded hydrogel demonstrated excellent blood compatibility, cell compatibility, and successfully promoted the healing of infected wounds in animal models [146].

Casein-based systems have shown great potential as carriers for the controlled delivery of antitumor drugs. Mehryab et al. [147] designed a casein-based hydrogel using an acid-induced gelation technique to achieve a controlled release of crocin, the primary carotenoid in saffron, known for its multiple therapeutic benefits, particularly its strong anti-tumor effects across various tissues and organs [148]. These hydrogels exhibited non-Newtonian pseudoplastic behavior. Among the different formulations, the hydrogel with the lowest casein-to-crocin weight ratio (10:1) demonstrated the highest crocin loading capacity, the greatest swelling in simulated saliva fluid within the first hour, and the most significant crocin release (58.07% over 24 h). This enhanced release profile was attributed to the increased porosity of the hydrogel structure. Regardless of formulation differences, all casein-based hydrogels enabled a sustained in vitro release of crocin [147]. In combination with alginate, casein has been used to incorporate green-synthesized selenium nanoparticles, which have known anticancer properties. The unique amalgamation enhances targeted cancer therapy by promoting sustained release of therapeutic selenium nanoparticles directly to tumor sites, thereby improving the therapeutic index while minimizing systemic toxicity. Casein’s amphiphilic nature and functional groups supported stable hydrogel formation with alginate and efficient encapsulation and retention of selenium nanoparticles, contributing to prolonged drug release and enhanced bioavailability at the cancer site. The green synthesis of the nanoparticles within this casein-alginate hydrogel reduced harmful chemical residues, aligning with eco-friendly and biocompatible treatment goals. Overall, this platform leverages casein hydrogel’s natural properties and selenium’s anticancer efficacy to offer a promising, safer, and effective targeted therapy strategy for cancer treatment [149].

### 6.5. Whey Protein

Whey proteins, derived from milk, exhibit outstanding functional, biological, and nutritional properties, making them highly suitable for various biomedical and food applications. The primary constituents of whey proteins are globular proteins, including β-lactoglobulin (β-LG), α-lactalbumin, and BSA [150]. Extensive research has demonstrated the potential of whey protein-based products, such as whey protein isolate (WPI) and whey protein concentrate (WPC), as well as some specific components, in the development of protein-based hydrogels. These hydrogels have been widely investigated for their ability to encapsulate and deliver bioactive molecules and micronutrients. Their formulation can be achieved using whey proteins in their native or aggregated states (such as fibrils and microgels), either alone or in combination with other materials, enhancing their functional versatility [151]. Whey protein amyloid fibrils, for example, have been used to create composite hydrogels for effective drug delivery, providing a robust and biofunctional scaffold due to their highly ordered beta-sheet-rich structure, which enhances hydrogel mechanical strength and stability. In this system, the whey protein fibrils were combined with gliadin nanoparticles to form a hybrid hydrogel matrix that exhibits tunable viscoelastic properties, allowing controlled encapsulation and release of hydrophobic bioactive compounds like curcumin. The nanostructured fibrillar network improved the hydrogel’s ability to sustain drug release while protecting curcumin from degradation. This approach demonstrated how whey protein-based hydrogels can be engineered at the nanoscale to optimize drug loading, release kinetics, and structural integrity, making them promising platforms for delivering poorly soluble therapeutic agents in biomedical applications [152].

WPI is a more purified form, containing 90% or more protein, obtained through additional filtration or ion-exchange steps that remove most of the fat and lactose. As a result, WPI has a cleaner composition and is suitable for applications requiring high protein purity, including clinical nutrition, sports supplementation, and biomaterial development. In research, particularly in the formulation of protein-based hydrogels or drug delivery systems, WPI is often preferred over whey concentrate because its higher purity and consistent composition improve the reproducibility of gelation behavior, mechanical properties, and functional performance [153]. Moreover, WPI has been widely investigated due to its ability to form long and slender protein nanofibrils (PNFs) with well-defined morphologies that frequently display strong interactions with their surroundings, resulting in the formation of hydrogels with potential for diverse applications, including biomedical, optoelectronic, and hybrid organic–inorganic materials, as well as templates for nanowire fabrication. These fibrils typically exhibit a width of approximately 4 nm and can extend to several micrometers in length. In comparison, other proteins, such as meat hemoglobin, rice globulin, and SPI, tend to produce shorter fibrils with less well-defined structures [61,154].

#### Drug Delivery Applications and Cancer Treatment

Whey protein-based hydrogels have demonstrated a remarkable capacity for releasing probiotics in the gastrointestinal tract. Whey protein hydrogels exhibit tunable viscoelasticity, pH- and temperature-responsive swelling, and efficient encapsulation properties due to the availability of reactive amino groups that enable physical or enzymatic crosslinking. These attributes make them effective in controlling the release of bioactives and sensitive therapeutics, similar to their role in safeguarding probiotics against gastrointestinal degradation. Moreover, whey protein hydrolysates, rich in bioactive peptides, can enhance the release kinetics and bioavailability of encapsulated drugs through improved solubility and degradability. The findings revealed that the survival rate of probiotics encapsulated within these hydrogels exceeded 96%, in stark contrast to the mere 37.43% survival rate observed for free probiotics [155]. In a separate study, Feng et al. developed an innovative double-layered encapsulation system for Lactobacillus plantarum, which significantly improved the thermal stability and tolerance of the probiotics in simulated gastrointestinal conditions [156]. The incorporation of BSA into β-LG amyloid fibril-based hydrogels demonstrates a tunable approach to modulating the structural and functional properties of whey protein hydrogels for drug delivery. BSA enhances β-LG fibrillogenesis and stabilizes the fibrillar network against pH-induced disruption, leading to a more compact and homogeneous microstructure. However, its intercalation between fibril chains may reduce mechanical strength by limiting fibril entanglement. In drug release studies using riboflavin as a model compound, release kinetics were governed by fibril content and BSA-Riboflavin binding affinity. Under enzymatic digestion, the degradation of BSA and the hydrogel matrix diminished the contribution of binding affinity, leaving fibril density as the dominant factor controlling release. Overall, BSA acts as a modulatory component within β-LG-based hydrogels, enabling fine control over network architecture, mechanical integrity, and release behavior, highlighting a versatile strategy for tailoring whey protein hydrogels in controlled delivery systems [157]. In combination with gelatin, WPI has been used to encapsulate and deliver N-acetylneuraminic acid (NeuAc), a bioactive compound closely related to sialic acids, for drug delivery applications. WPI’s excellent biocompatibility and gelation properties are leveraged to enhance mechanical strength, encapsulation efficiency, and controlled-release behavior within the hydrogel network. The hydrogel’s drug release profile can be tuned by modifying the gelatin-to-WPI ratio, enabling pH-responsive and sustained release patterns particularly suited for oral administration. This combination allows the hydrogel to protect the bioactive compound as it passes through the gastrointestinal tract and then facilitates controlled, site-specific release in response to intestinal pH. The approach validates the potential of whey-protein-based hydrogels as a versatile platform for drug delivery, promoting improved bioavailability and stability of loaded compounds during gastrointestinal transit [158].

WPI hydrogels have also been explored for the delivery of therapeutic compounds targeting tumor treatment. Recent studies have shown that WPI-based hydrogels can effectively encapsulate hydrophobic antitumor molecules such as cannabidiol (CBD), enabling controlled, pH-responsive release within gastrointestinal regions commonly affected by cancers like colorectal cancer. A central component of this work involved assessing hydrogel performance under simulated digestive conditions to determine their suitability as drug-delivery platforms for CRC. Release profiling and cell-viability assays confirmed that CBD preserved its biological activity after encapsulation, underscoring the protective role of the WPI matrix. Notably, the hydrogels remained stable in stomach-like environments, withstanding acidic pH and enzymatic degradation to prevent premature CBD release. In contrast, both matrix degradation and CBD release increased markedly under intestinal conditions, supporting their potential for targeted delivery [159]. Furthermore, previous research has shown that CBD exerts antiproliferative effects on HT29 CRC cells in vitro [160]. Therefore, to assess the efficacy of WPI-CBD hydrogels in impacting cell viability, HT29 cells were treated for 72 and 96 h. While no significant effects were observed at 72 h, by 96 h, a notable reduction in cell viability was recorded—20.4% (WPI-CBD2), 18.3% (WPI-CBD4), and 21.8% (WPI-CBD5) (*p* < 0.05). Given these results, in combination with the observed bioactivity retention, the study concluded that WPI hydrogels hold strong potential as an effective delivery platform for hydrophobic molecules in CRC treatment. These findings support the continued investigation of WPI-CBD hydrogels as a promising candidate for CRC therapy [159].

The integration of tannic acids (TAs) into biomaterials has been associated with reduced tumor necrosis factor levels [161] and suppression of inflammatory cytokines, motivating their use in anticancer applications [162]. Building on these properties, Mayorova et al. developed pH-sensitive, cytocompatible hydrogels that combine TAs with whey protein isolate (WPI). Two TA derivatives. Polygalloyl glucoses (ALSOK 02) and polygalloyl quinic acids (ALSOK 04) were compared, differing in molecular weight and structure. The hydrogels exhibited marked pH-responsive behavior: at acidic pH (5), significant TA release occurred due to protein dissociation and protonation. This is particularly relevant for cancer therapy, as both tumor microenvironments and endosomal compartments are typically more acidic than healthy tissues. The pH-dependent release profile supports their suitability as localized scaffolds for gastrointestinal cancers, where pH ranges from acidic to basic. Cytotoxicity studies using Hep-2 laryngeal cancer cells revealed minimal effects for WPI-only hydrogels, while TA-containing formulations significantly reduced metabolic activity in a concentration-dependent manner. Hydrogels with the highest TA/WPI ratio decreased cell viability by 50% at 24 h and 80% at 48 h. Overall, WPI hydrogels incorporating TAs (3 mg/mL and TA/WPI ratio 0.075) were identified as the most promising formulation for sustained anticancer activity [163]. Together, these findings underscore whey-protein-based hydrogels as a highly versatile and tunable platform capable of protecting sensitive bioactives, enabling targeted and sustained release, and supporting promising therapeutic applications ranging from probiotic delivery to localized anticancer treatment.

### 6.6. Collagen

Collagen is a key structural protein found in the bodies of vertebrates and is the most abundant protein in mammals, constituting about 20–30% of total body protein. Its fundamental structure consists of tropocollagen, which comprises three intertwined strands that form a triple helix, interconnected by various non-covalent bonds. Ultimately, collagen is formed through covalent crosslinking of tropocollagen molecules [164]. Due to its excellent biocompatibility, minimal immune system stimulation, and biodegradability, collagen is extensively utilized in medical applications [165].

Collagens, especially type I collagen, account for approximately 90% of the protein found in human connective tissues and are commonly utilized to create single-component hydrogels [166]. At a neutral pH, collagen fibrils can self-assemble into bundled fibers, which can crosslink to form matrices. When combined with a water-based solvent system, these matrices ultimately develop into hydrogels [167]. Collagen is a key structural protein found in the ECM of vertebrates, present in tissues such as skin, connective tissue, bone, and cartilage. Due to its biological origin, collagen has low immunogenicity, meaning it is unlikely to trigger significant inflammation or immune responses when introduced into body tissues [168]. Collagen hydrogels mimic the ECM’s structure, making them closely resemble natural biological tissues. They can be degraded by collagenase within the body, allowing them to fully utilize their biological functions. Current methods for preparing collagen hydrogels include self-assembly and chemical crosslinking [169]. Collagen self-assembly involves the organization of collagen fibers, where molecules connect end-to-end in a staggered manner, trapping solvent within the structure and preventing free flow. This method primarily relies on noncovalent interactions, which can result in suboptimal performance. In contrast, chemically crosslinked collagen hydrogels are created through covalent bonding, often employing small-molecule aldehydes or epoxides as crosslinking agents. While these chemically crosslinked hydrogels exhibit good elasticity, they may lack the softness found in hydrogels produced via self-assembly [170]. Collagen hydrogels are often injectable under physiological conditions and can be combined with other biomolecules, like hyaluronic acid, to enhance their properties for applications in cartilage, bone, and skin regeneration [171]. Recent advances include multifunctional collagen hydrogels endowed with antibacterial, conductive, or antioxidant functionalities to improve wound healing and tissue repair outcomes [172,173].

The concentration of collagen and the degree of crosslinking significantly influence the hydrogel’s stiffness, porosity, and biological performance, making these materials highly versatile and customizable for various regenerative medicine and drug delivery systems [174]. This versatility, combined with collagen’s inherent biological advantages, underscores its widespread use in medical fields such as tissue engineering, wound healing, and controlled drug release.

#### Drug Delivery Applications and Cancer Treatment

Collagen-based hydrogels have emerged as highly promising biomaterials for drug delivery, offering a unique combination of biocompatibility, tunable structure, and physiological relevance that enables precise and efficient therapeutic release [175]. Biocompatible hydrogels comprising collagen, chitosan, and polyurethane have shown significant potential for wound healing and controlled drug release, particularly leveraging the properties of collagen as a core biomaterial. Collagen forms a natural, biodegradable, and biocompatible matrix that mimics the extracellular environment, promoting cell adhesion, proliferation, and tissue regeneration. When combined with chitosan and polyurethane, these hydrogels exhibit enhanced mechanical strength, stability, and antibacterial properties due to chitosan’s intrinsic antimicrobial effects. These collagen-based hydrogels demonstrated controlled release profiles, exemplified by the release of ketorolac (a non-steroidal anti-inflammatory drug) under physiological conditions (pH 7, 37 °C). This controlled release capacity, combined with the biological activity of the hydrogel, supports its use as a smart wound dressing material that not only protects the wound but also provides localized, sustained therapeutic delivery to aid healing [176]. The versatility of collagen-based hydrogels is further demonstrated in complex regenerative tasks, such as peripheral nerve repair. A recent study reported a collagen–reduced graphene oxide (COL–rGO) biocomposite hydrogel synthesized via horseradish peroxidase/hydrogen peroxide–mediated crosslinking, yielding an injectable, mechanically reinforced, and cytocompatible network with controlled degradation. The COL–rGO matrix supported fibroblast adhesion and proliferation in vitro and promoted wound-healing–related marker expression. In vivo, it accelerated wound closure, enhanced re-epithelialization, and increased collagen deposition compared with pristine collagen gels, demonstrating that conductive nanofillers and mild enzymatic crosslinking can significantly enhance the bioactivity of collagen hydrogels for chronic wound treatment [32].

Fan et al. [177] engineered a hydrogel system using collagen derived from tilapia skin combined with chitosan (HCC) to explore its efficacy as a carrier for therapeutic nanobodies. Their investigation centered on two nanobodies: 2D5, which specifically binds to carcinoembryonic antigen (CEA), and KPU, targeting programmed death-ligand 1 (PD-L1). Among the different formulations, HCC10, comprising equal concentrations (10 mg/mL) of collagen and chitosan, displayed the highest stability. To evaluate controlled nanobody release, they examined the hydrogel’s behavior across varying pH levels, reflective of physiological conditions: human skin (pH 5.5), tumor microenvironment (pH 6.8), and bodily fluids (pH 7.4) [178,179]. Over 168 h, cumulative 2D5 release reached 68.3% at pH 5.5, 56.4% at pH 6.8, and 28.4% at pH 7.4. KPU, however, demonstrated a consistent release profile with 45.1%, 46.5%, and 44.9% at these pH levels. These results indicate that 2D5 exhibits pH-sensitive release, aligning with tumor environments, while KPU maintains steady dispersion, underscoring the hydrogel’s ability to modulate nanobody delivery based on pH variations and, therefore, its potential in targeted cancer therapy [177]. Calcium carbonate microspheres loaded with an iron and selenopeptide conjugate, incorporated into a human-like collagen scaffold, have been used to form a multifunctional collagen-based hydrogel designed for both skin filling and gastric cancer therapy. This collagen scaffold enabled precise and sustained delivery of the therapeutic iron and selenium conjugates directly to the tumor site, reducing systemic toxicity while promoting cancer cell death through apoptosis and ferroptosis mechanisms. The hydrogel’s integrated design combined the mechanical properties of calcium carbonate microspheres with the bioactivity of the conjugate and collagen’s natural compatibility, allowing effective localized skin regeneration and potent antitumor effects in gastric cancer treatment [180]. Moreover, such collagen-based hydrogels exhibited controlled degradation and responsive drug release, making them promising platforms for advanced, minimally invasive cancer therapies with improved therapeutic efficacy and reduced side effects compared to systemic chemotherapy [181]. Recent research has explored incorporating immune checkpoint inhibitors or immunostimulatory agents into hydrogels to suppress cancer metastasis [182]. Hwang et al. [183] developed a collagen-based thermosensitive hydrogel (pTRG) for immunotherapeutic agent delivery. The hydrogel was loaded with indocyanine green and polyinosinic:polycytidylic acid (poly I:C), an immune stimulator. Its therapeutic efficacy was evaluated in CT-26 carcinoma and 4T1 lung metastasis mouse models. When combined with photothermal therapy (PTT) and immunotherapy, pTRG promoted tumor-associated antigen expression, amplifying immune responses. Upon near-infrared irradiation at 808 nm, the hydrogel liquefied at 60 °C, ensuring controlled release of poly I:C, STING ligand, and immune checkpoint inhibitors such as anti-PD-1 and anti-PD-L1 antibodies [184]. Drug quantification involved measuring differences between initially incorporated amounts and post-irradiation residuals. The hydrogel effectively eradicated CT-26 cancer cells in the lungs. Additionally, in re-challenge experiments with 4T1 cells, mice pre-treated with pTRG successfully resisted lung infiltration, whereas untreated mice exhibited metastasis. These findings highlight pTRG’s potential in integrating photothermal therapy with immunotherapy for breast cancer treatment [183].

### 6.7. Elastin

Elastin is a vital structural protein predominantly found in the ECM of various tissues, particularly within arterial walls, where it imparts elasticity and resilience, enabling blood vessels to withstand fluctuations in blood pressure. Beyond the vascular system, elastin is abundantly present in the lungs, skin, and ligaments, playing a fundamental role in maintaining tissue integrity and mechanical flexibility [185]. Elastin is primarily composed of tropoelastin monomers, which undergo extensive covalent cross-linking to form mature elastic fibers. This cross-linked network is responsible for elastin’s unique elastic properties and long-term durability in vivo [186]. Elastin fibers are typically associated with microfibrils, which act as a scaffold, further enhancing their mechanical stability and contributing to skin elasticity and resilience against mechanical deformation [187]. The development of elastin-like polymers (ELPs) and elastin-like recombinamers (ELRs), inspired by the repetitive motifs found in native elastin, represents a significant advancement in biomaterials science. These recombinant biopolymers, produced through genetic engineering techniques, closely replicate the physicochemical characteristics of natural elastin. Their biocompatibility and low immunogenicity render them highly suitable for biomedical applications, including drug delivery and tissue engineering [188]. A key feature of ELRs is their capacity for self-assembly, wherein individual polypeptide units autonomously organize into well-defined nanostructures, such as nanoparticles, hydrogels, and fibrous scaffolds. This self-assembling behavior is driven by reversible phase transitions in response to environmental stimuli, such as temperature, ionic strength, or pH [189]. Furthermore, ELPs and ELRs can be precisely engineered through genetic design, enabling control over their molecular weight, amino acid sequence, and structural properties. This molecular-level tunability facilitates the creation of monodisperse polymers with customizable pharmacokinetics and enhances the stability and solubility of therapeutic agents. These recombinant elastin-based materials can be functionalized with bioactive molecules, drugs, or targeting ligands, providing a versatile platform for the development of site-specific drug delivery systems [190]. In a recent study, ELR-based hydrogels have demonstrated significant potential in modulating post-ischemic remodeling in models of myocardial infarction (MI), particularly in non-transmural MI in sheep. Recent studies highlight that injectable, degradable ELR hydrogels, which mimic the native ECM, can reduce fibrosis, promote angiogenesis, and preserve cardiomyocyte integrity in the border zone of the infarcted tissue [191]. When applied in a clinically relevant ovine model, these hydrogels facilitated functional cardiac recovery by favorably influencing the ischemic environment, attenuating adverse remodeling, and fostering tissue regeneration. The elastic and biocompatible properties of ELR hydrogels enable them to integrate seamlessly into cardiac tissue, while their tunable degradation profiles allow controlled release of therapeutic agents and support tissue healing processes. Moreover, the ability of these hydrogels to mimic the ECM and exert mechanical stabilization within the infarcted myocardium underpins their effectiveness in improving cardiac function and preventing heart failure post-MI [192]. Their unique capacity to modulate cellular responses and remodeling pathways positions ELR hydrogels as promising candidates for regenerative therapy in post-ischemic cardiac repair [191]. The structural versatility and biological compatibility of elastin-derived materials position them as promising candidates for next-generation biomaterials in regenerative medicine, controlled drug delivery, and tissue remodeling applications [47].

#### Drug Delivery Applications and Cancer Treatment

**Figure 5:**
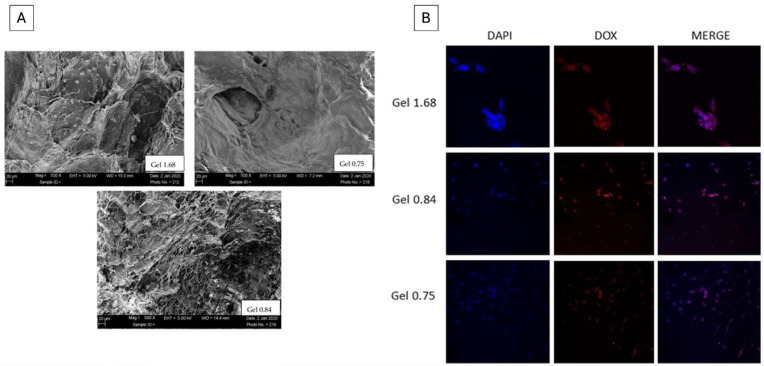
Morphological and biological characterization of ELP hydrogels: (**A**) SEM images of freeze-dried composites showing scaffold morphologies (examined at 5 kV, sputter-coated with Au/Pd for 1.5 min at 9 V); (**B**) Qualitative co-localization of DAPI (blue) and doxorubicin (red) in U-87 MG cells treated with ELP hydrogels, visualized via confocal microscopy to establish nuclear and perinuclear localization of the drug [200]. Reproduced with permission from “© 2022 by the authors. Licensee MDPI, Basel, Switzerland”.

Elastin-derived materials have been investigated in various studies as innovative drug delivery platforms, including their application in the targeted delivery of salvianolic acid B for the treatment of MI [193]. Derived from elastin-mimetic peptides, this hydrogel combined natural elasticity, biocompatibility, and tissue-like mechanical resilience, enabling it to adapt to cardiac movement and maintain structural integrity after injection. The dynamic covalent or reversible physical interactions within the peptide network imparted self-healing behavior, allowing the hydrogel to recover from mechanical disruptions and ensure continuous drug release [194]. Salvianolic acid B, a potent antioxidant and cardioprotective compound, benefits from encapsulation in the elastin matrix, which provides localized and prolonged delivery to the infarcted region, enhancing myocardial repair, angiogenesis, and inhibition of oxidative stress. The hydrogel’s injectable nature facilitated minimally invasive administration, while its structural tunability allowed precise control over degradation rate and release kinetics, making elastin-based self-healing hydrogels a highly effective and biomimetic strategy for cardiac tissue regeneration and post-infarction drug therapy [193]. ELR hydrogels have emerged as promising biomimetic platforms for developing 3D breast cancer models. Blanco-Fernandez et al. [195] constructed ELR hydrogels using two distinct recombinant polypeptides, each composed of repeating pentapeptide sequences VPGXG (where V is valine, P is proline, G is glycine, and X is any amino acid other than proline). One ELR variant was engineered to incorporate matrix metalloproteinase (MMP)-sensitive domains, reflecting the proteolytic environment characteristic of breast tumors, along with cyclooctyne groups for click-chemistry crosslinking [196,197]. The second ELR was functionalized with arginylglycylaspartic acid (RGD) motifs to promote integrin-mediated cell adhesion, alongside azide groups enabling hydrogel formation through click-chemistry [198]. These ELR hydrogels closely mimic the ECM properties of breast tumors, offering a physiologically relevant microenvironment for in vitro cancer studies. The potential of these hydrogels was evaluated by encapsulating non-tumorigenic MCF10A cells, non-invasive MCF7 breast cancer cells, and invasive MDA-MB-231 breast cancer cells within the 3D hydrogel matrix. Upon exposure to various concentrations of the chemotherapeutic agent DOX, cells encapsulated within ELR hydrogels exhibited increased drug resistance compared to conventional 2D cultures, with the MCF7 cell line showing particularly pronounced resistance [199]. This observation underscores the capacity of ELR hydrogels to more accurately recapitulate the tumor microenvironment, thus providing a robust platform for assessing chemotherapeutic responses and enhancing the predictive value of preclinical cancer models [195]. Recent research highlights the potential of elastin-like polypeptide hydrogels in advancing cancer therapy through localized drug delivery. One notable study developed ELP hydrogels for tunable, sustained local chemotherapy in malignant glioma, demonstrating their capacity to release chemotherapeutic agents like DOX over extended periods, thereby enhancing treatment efficacy while minimizing systemic toxicity. The internal architecture of these ELP composites, as revealed by SEM analysis (Figure 5A), exhibited a highly defined scaffold morphology characterized by a porous network essential for controlled drug loading. These hydrogels were engineered for precise control over drug release profiles by manipulating polymer composition, making them suitable for post-surgical applications in glioma management. The biological performance of this system was further validated through co-localization studies using confocal microscopy. As shown in Figure 5B, the treatment of U-87 MG cells with DOX-loaded ELP hydrogels resulted in a clear co-localization of the drug with the DAPI-stained nuclei. This qualitative visualization of nuclear and perinuclear fluorescence confirms the effective intracellular delivery and localization of doxorubicin, highlighting the potential of ELP hydrogels to serve as effective reservoirs for targeted chemotherapy in neuro-oncology [200].

In addition, another key investigation introduced an engineered elastin-like polypeptide-based hydrogel designed for co-delivery of chemotherapeutics and immune checkpoint inhibitors such as PD-L1 antibodies. This hydrogel depot facilitates in situ delivery, promoting a robust immune response, and significantly potentiates cancer immunotherapy by increasing tumor-infiltrating CD8^+^ T cells and depleting regulatory T cells (Tregs), thus improving overall treatment outcomes in melanoma and other tumor models [201]. Collectively, these elastin-based biomaterials demonstrate considerable potential as multifunctional platforms for localized, controlled, and sustained delivery of different biomolecules and anticancer agents.

An overview of representative protein-based hydrogels and their applications is presented in Table 3.

### 6.8. Peptide-Based Hydrogels

In addition to full-length proteins, short peptide sequences have emerged as versatile building blocks for hydrogel design, offering highly tunable biochemical and mechanical properties. Their sequence-defined nature allows rational control over self-assembly, degradability, and interactions with therapeutic molecules or cancer cells. These features make peptide-integrated hydrogels particularly attractive for developing smart delivery systems capable of responding to tumor-specific cues [202,203]. Peptide-based supramolecular hydrogels, synthesized from the dehydration condensation of natural amino acids, present several advantages over other biomaterials due to their straightforward synthesis, ease of modification, tunable mechanical properties, biocompatibility, and biodegradability. These hydrogels possess inherent bioactivity and can be readily functionalized through simple chemical modifications to achieve diverse applications [204]. For example, self-assembling peptide hydrogels have been used to provide a dynamic platform for creating dual gradients of mechanical stiffness and biochemical signals to guide osteogenic cell behavior. In this system, enzymatic crosslinking by TGase formed covalent bonds between glutamine and lysine residues within the peptide network, stabilizing the hydrogel structure while allowing spatial modulation of crosslinking density. By controlling the local enzymatic activity and peptide composition, gradients in mechanical rigidity and bioactive ligand presentation can be established within a single hydrogel matrix. These dual gradients synergistically influence MC3T3-E1 preosteoblast cell adhesion, proliferation, and differentiation by mimicking the complex microenvironment of developing bone tissue [205]. Peptide-based hydrogels with immune-instructive physiochemical properties could also create a supportive environment that reduces inflammation and promotes nerve regeneration. By mimicking the natural ECM and modulating immune responses, these hydrogels enhanced neural cell survival and neurite growth, helping to calm nerves and aid functional recovery after injury [206]. Notably, peptide-based hydrogels address common drawbacks associated with traditional chemotherapeutic drugs, such as poor water solubility, non-selective distribution, and short biological half-life [207]. Xu et al. devised a paclitaxel-peptide conjugate capable of forming a supramolecular hydrogel, demonstrating anticancer properties. By tailoring the hydrophilic and hydrophobic sequences of peptides, drug-peptide conjugates can be rationally designed to facilitate site-specific drug release [208]. For instance, ester, amide, or disulfide bonds can link drugs to peptides, enabling the formation of self-delivery hydrogels that allow for high drug-loading capacity and stimuli-responsive drug release [209]. Zhang et al. developed a methotrexate (MTX)-peptide conjugate incorporating a pH-sensitive motif (2,3-dimethylmaleic anhydride), which transitions from sol to gel under the mildly acidic conditions of the tumor microenvironment, enhancing drug retention and therapeutic efficacy in tumor-bearing mice [210]. Xu et al. introduced a curcumin-peptide conjugate (CurcuminFFE-CS-EE), which combines supramolecular nanomedicine with radiation therapy. This conjugate undergoes glutathione (GSH)-mediated reduction, leading to hydrogel formation. Both in vitro and in vivo data suggest that this radiosensitizer enhances CRC cell sensitivity to radiation and effectively inhibits tumor growth [211]. Similarly, Hu et al. designed an amphiphilic peptide (C16-GNNQQNYKD-OH, or C16N) capable of co-assembling with losartan into a hydrogel. This co-assembled hydrogel achieved sustained drug release for up to nine days in vitro and showed efficacy in suppressing tumor growth and lung metastases in vivo [212].

Moreover, a pH-responsive ionic-complementary peptide was formulated into a supramolecular hydrogel through self-assembly with DOX. Upon exposure to the acidic tumor microenvironment, the hydrogel disassembles, transitioning from nanofibers to nanospheres, thereby enhancing cellular uptake and improving antitumor efficacy [213]. Wang et al. devised an indomethacin-peptide conjugate (IDM-FFpYSV) capable of self-assembling into nanofiber hydrogels in the presence of cancer cell-expressed alkaline phosphatase. This hydrogel facilitated sustained indomethacin release and showed significant tumor suppression in HeLa xenografts [214]. In another study, Wang et al. created a peptide hydrogel composed of (RADA)4 and an octaarginine (R8) sequence. The (RADA)4 segment drives nanofiber formation, while R8 enhances cell membrane penetration and drug retention by reducing efflux associated with multidrug resistance, offering a potent therapeutic platform [215]. Wu et al. proposed a combinatorial approach that merges chemotherapy with photodynamic therapy (PDT). They synthesized a lonidamine-peptide conjugate (LND-peptide), which targets mitochondria and co-assembles with the anionic photosensitizer TPPS4 through electrostatic interactions. This hybrid hydrogel increased ROS production, depleted ATP, and disrupted mitochondrial function, thereby sensitizing cancer cells to PDT. In vitro and in vivo results demonstrated robust antitumor effects against A375 melanoma cells, illustrating the potential of this integrated chemotherapy-PDT platform [216]. Mei et al. developed RGD-derived peptide conjugates (GFFYGRGDHn, with n = 0–2, labeled 1-RGD, 1-RGDH, and 1-RGDHH) that co-assemble into hydrogels in the presence of DOX. The interaction between the peptide and the drug strengthens the hydrogel network via electrostatic forces, reducing the minimum gelation concentration and enhancing mechanical stability. These DOX-loaded hydrogels exhibited pH-responsive drug release, with increased release in acidic conditions (pH 6.5–5.5), corresponding to tumor environments. Additionally, they demonstrated superior cytotoxicity against A549, MCF-7, and HeLa cancer cells compared to free DOX, highlighting their potential in targeted drug delivery [217]. Karavasili et al. explored the Ac-(RADA)4-CONH2 peptide hydrogel for localized co-delivery of DOX and curcumin (CUR). This system effectively induced apoptosis in glioblastoma cells in vitro [218]. In subsequent studies, the same authors investigated its application for head and neck cancer therapy. Drug release studies revealed a rapid DOX release within four days, while CUR-loaded hydrogels exhibited sustained drug release over 20 days. Compared to free drugs, the peptide-based hydrogels enhanced cellular uptake and cytotoxicity, particularly in formulations with CUR, which induced higher early apoptosis levels. These findings suggest that Ac-(RADA)4-CONH2 hydrogels serve as promising delivery systems for both hydrophilic and hydrophobic chemotherapeutic agents [219].

Leach et al. proposed the use of peptide-based hydrogels for the controlled delivery of synthetic cyclic dinucleotides (CDNs) to enhance antitumor immune responses in preclinical models. A self-assembling peptide nanofiber hydrogel with the sequence K2(SL)6K2 (MDP) demonstrated a significantly slower release rate of CDNs, approximately eight times lower, compared to conventional collagen hydrogels. This sustained release system effectively minimized infiltration into normal cells in a localized delivery model of head and neck squamous cell carcinoma (HNSCC). In experiments conducted on wild-type C57BL/6 mice, a single injection of the peptide hydrogel resulted in controlled CDN release, highlighting its potential as a targeted delivery platform for cancer immunotherapy [220]. Luo et al. designed a hybrid peptide hydrogel (Nap-GFFY), produced via a heating–cooling process, capable of encapsulating ovalbumin (OVA) antigens. This antigen-loaded hydrogel induced potent CD8+ T-cell responses, enhancing immunotherapy in a tumor-bearing mouse model [221]. Lv et al. developed a peptide hydrogel co-loaded with DOX and cytokines (IL-2/IFN-γ). This hydrogel facilitated sustained release, serving as a chemo-immunotherapy platform for melanoma treatment [222]. Cao et al. constructed a mitochondria-targeting peptide (TpYCR) that co-assembled with DOX into nanoparticles, subsequently transitioning into nanofibers and hydrogels in response to alkaline phosphatase and GSH in the tumor microenvironment. This transformation prolonged DOX retention, amplifying the immune response and improving antitumor efficacy [223]. Table 4 reports a summary of recently used peptide-based hydrogels as drug delivery systems.

While peptide-based hydrogels show promise in drug delivery and cancer therapy, challenges remain, including ensuring stability in vivo, minimizing immunogenic responses, and achieving precise, site-specific drug release without off-target effects. Addressing these limitations will be crucial for the clinical translation of peptide-based supramolecular hydrogels.

## 7. Conclusions and Future Perspectives

Protein-based hydrogels represent one of the most dynamic and versatile classes of biomaterials, positioned at the forefront of biomedical innovation due to their inherent biocompatibility, structural similarity to native extracellular matrices, and the remarkable tunability of their physicochemical properties. Their multifunctional nature, arising from the diverse chemical functionalities and hierarchical architectures of proteins, allows these hydrogels to form highly effective platforms for localized, sustained, and stimuli-responsive drug delivery. Over the past decade, significant progress has been made in refining synthesis approaches, from precision chemical modification and enzymatic crosslinking to emerging bio-orthogonal chemistries, as well as in integrating external triggers such as pH, temperature, redox gradients, and enzymatic activity to achieve responsive and on-demand release profiles. Importantly, several protein-based hydrogels, including fibrin, collagen, and gelatin systems, have already reached clinical use, demonstrating regulatory feasibility and safety. Nevertheless, several critical challenges continue to limit the full clinical translation of these hydrogel systems. Achieving mechanical robustness without compromising biocompatibility or dynamic network behavior remains a major hurdle, particularly for applications requiring long-term implantation or mechanical load-bearing. Additionally, maintaining the bioactivity of embedded therapeutics (ranging from small molecules to fragile biologics such as peptides, growth factors, and nucleic acids) requires more sophisticated encapsulation strategies that protect cargo during gelation and throughout the release process. Equally important is the need to fine-tune drug release kinetics with high precision to match the intricate spatiotemporal demands of complex biological microenvironments, including inflamed, fibrotic, or tumor tissues.

The standardization of natural raw materials remains a primary bottleneck; the inherent biological variability of animal-derived proteins complicates the establishment of Current Good Manufacturing Practice (cGMP) compared to synthetic alternatives. Furthermore, sterilization stability is a major concern, as conventional methods like autoclaving or gamma radiation can denature protein secondary structures, altering both the mechanical robustness and the drug release kinetics. Additionally, the immunogenic potential of non-human proteins (such as silk or plant-derived proteins) requires rigorous long-term safety profiling to ensure no adverse inflammatory responses occur in human patients.

From a translational standpoint, we believe that protein-based hydrogels are well-positioned to enter clinical testing, but their success will be highly indication-driven rather than universal. Systems that exploit their biological fidelity, injectability, and localized action, particularly in wound healing, regenerative medicine, and local drug delivery, are likely to progress more rapidly through clinical evaluation than highly complex, multifunctional constructs. The main bottleneck may not be conceptual innovation but reproducibility, manufacturability, and regulatory alignment. Simplifying hydrogel designs without sacrificing key biological functions, adopting scalable and well-controlled production strategies (preferably using recombinant proteins), and defining clear clinical use cases early in development will be decisive for successful translation.

A critical benchmark for the clinical relevance of protein-based hydrogels is the success of currently marketed products, which have paved the regulatory path for more complex delivery systems. While many “smart” hydrogels discussed in this review remain in the preclinical phase, first-generation protein matrices are already staples in surgical and wound-care settings. Collagen-based products represent the largest clinical category; for instance, Avitene™ (microfibrillar collagen) and Gelfoam^®^ (an absorbable gelatin sponge) are widely utilized as topical hemostats. Similarly, fibrin-based sealants, such as Tisseel, exploit the natural final stage of the coagulation cascade to achieve tissue adhesion and sealing in surgical procedures. In the specific context of oncology and systemic delivery, the clinical landscape is further validated by albumin-based technologies. Although not a traditional macroscopic hydrogel, Abraxane^®^ (albumin-bound paclitaxel) utilizes the biological transport properties of proteins to enhance drug accumulation in tumors, setting a precedent for the regulatory approval of protein-drug complexes. Furthermore, the development of recombinant human collagen (e.g., rhCollagen) and Silk-derived scaffolds for soft tissue augmentation is currently bridging the gap between basic research and clinical trials. These products address the “reproducibility gap” by utilizing biotechnological production methods that eliminate the batch-to-batch variability inherent in animal-sourced tissues, providing a clearer roadmap for the FDA approval of the next generation of multifunctional, stimuli-responsive protein hydrogels.

Looking ahead, the development of next-generation protein hydrogels will demand a rational and multidisciplinary design philosophy. Incorporating advanced features such as programmable degradation, dynamic and reversible crosslinking, and controllable viscoelasticity will provide new avenues for achieving highly customized therapeutic profiles. Integrating immunomodulatory cues, either through engineered protein sequences or the incorporation of immune-active components, holds particular promise for improving outcomes in regenerative medicine, autoimmune disorders, and oncology. Parallel efforts should explore hybrid hydrogel constructs that seamlessly combine natural proteins with synthetic polymers, bioactive peptides, nanoparticles, or supramolecular assemblies, thereby leveraging synergistic interactions to enhance mechanical performance, stability, and functional versatility. Ultimately, the future of protein-based hydrogel technology lies in collaborative, interdisciplinary research that unites materials science, polymer chemistry, molecular and cell biology, and clinical engineering. Such integrated efforts will accelerate the creation of customizable, safe, and clinically translatable hydrogel platforms capable of supporting the next generation of localized drug delivery systems and personalized therapeutic interventions.

## Figures and Tables

**Table 1:** Comparative analysis of animal-derived versus plant-derived proteins for hydrogel fabrication: balancing bioactivity, clinical translation, and sustainability.

Feature	Animal-Based (Collagen, Gelatin, Silk)	Plant-Based (Soy, Zein, Pea)
Primary Bioactivity	High: Inherent RGD sequences promote cell adhesion and signaling.	Lower: Primarily structural; often requires functionalization for cell-specific interaction.
Immunogenicity	Variable: Generally low, but potential for zoonotic pathogen transmission or foreign body response.	Minimal: No risk of animal-derived pathogens; generally hypoallergenic in systemic delivery.
Consistency	Poor: High batch-to-batch variability due to animal age, health, and extraction methods.	Excellent: High reproducibility due to standardized agricultural harvesting and processing.
Mechanical Control	High: Easy to tune stiffness via enzymatic or chemical crosslinking to mimic soft tissues.	Moderate: Often requires hybridization with synthetic polymers to reach high toughness.
Regulatory Path	Established: Long history of clinical use but involves rigorous screening for pathogens.	Developing: Gaining FDA “GRAS” (Generally Recognized as Safe) status, simplifying the path.
Sustainability	Low: High environmental footprint and ethical considerations in animal sourcing.	High: Sustainable, renewable, and cost-effective for large-scale pharmaceutical production.

**Table 2:** Summary of widely used protein-based materials for hydrogel production, highlighting their intrinsic properties, crosslinking strategies, and therapeutic potential in drug delivery systems.

Protein Source	Main Characteristics	Crosslinking Methods	Therapeutic Potential
Gelatin	Denatured collagen, polyampholyte with balanced cationic, anionic, and hydrophobic groups	Chemical, enzymatic (e.g., transglutaminase), physical (thermal)	Drug delivery, wound healing, immune stimulation
Collagen	Natural ECM protein, biocompatible, promotes cell adhesion and growth	Chemical (e.g., glutaraldehyde), enzymatic, physical (thermal)	Tissue engineering, wound healing, drug delivery
Silk Fibroin	High mechanical strength, slow degradation, biocompatible	Chemical (e.g., genipin), physical (shear), enzymatic	Controlled drug release, cancer therapy, neurotherapy
Whey Protein	Nutritionally rich, bioactive peptides, capable of gelation under heat	Enzymatic, heat-induced, chemical	Oral delivery, nutraceuticals, controlled drug release
Casein	Amphiphilic, self-assembling, good emulsifier	Chemical, enzymatic	Chemotherapeutic delivery, cancer treatment
Soy Protein Isolate	Plant-based, rich in essential amino acids, biodegradable	Chemical, enzymatic	Drug delivery, tissue scaffolds
Elastin	Elastic, resilient protein, supports cell adhesion	Chemical, enzymatic	Drug delivery, cancer treatment
Albumin	High binding capacity, biocompatible	Chemical, enzymatic	Drug carriers, targeted delivery
Keratin	Structural protein, supports cell attachment	Chemical, physical	Tissue engineering, wound healing
Fibronectin	Glycoprotein, involved in cell adhesion, growth	Chemical, enzymatic	Tissue regeneration, drug delivery

**Table 3:** Overview of protein-based hydrogel systems for drug delivery: protein source, synthesis methods, loaded agents and experimental outcomes.

Protein Source	Synthesis Mechanism	Loaded Drug	In Vitro/In Vivo Outcomes	References
Gelatin (phenylboronic acid-modified)	UV crosslinking (boronic ester formation)	Epigallocatechin gallate (EGCG)	In vivo: Reduced inflammation, maintained disk structure, improved cell protection	[94]
Gelatin-guar gum	Schiff base crosslinking with oxidized guar gum	Antibiotics, general drugs	In vitro: Strong antibacterial (MRSA), excellent cytocompatibility, adhesive/self-healing, non-irritant	[95]
Gelatin Methacrylate (GelMA) + NBNAGA	UV/photo-crosslinking; nitrobenzyl chemistry	Doxorubicin, Rifampicin	In vitro: Controlled/triggered release, antibacterial; In vivo: Improved wound healing, on-demand release	[98]
Gelatin + Pluronic micelles	Injectable composite dual-encapsulation	Curcumin, 5-FU	In vitro: One-month sustained 5-FU release; strong cytotoxicity on HT-29 cells, synergy in dual-drug setup	[99]
Gelatin-AA/AMPS	Chemical crosslinking with acrylic acid	OXP	In vitro: Dose-dependent cytotoxicity in Vero, MCF-7, HCT116 cells; In vivo: Improved efficacy, less systemic side effects	[100]
SF + Sericin + PCL	Physical blend, porogenic process	Diclofenac sodium (model drug)	In vitro: Swelling, sustained release; antibacterial activity; suitable for wound healing	[114]
BMSF/AASF	Blended silk fibroins, injectable hydrogel	DOX, DEX	In vitro: Slow DOX release, strong cytotoxicity for MDA-MB-231 cells, promising in 3D tumor models, tissue regeneration	[122]
SPI-HEMA	Grafting (HEMA), pH-sensitive mechanisms	Paracetamol (model)	In vitro: pH-responsive, controlled release, promotes cell adhesion and viability	[131]
SPI with LIPO-DOX	In situ-gelling with doxorubicin-loaded liposomes	DOX	In vitro: High loading (>90%), gradual release, reduces cell viability of U87-MG glioblastoma cells in dose-dependent fashion	[135]
Casein-γ-PGA	Enzymatic crosslinking (mTGase), biodegradable network	Vitamin B12, Aspirin	In vitro: Controlled dual-release, stable in mild conditions, suitable for multiple drug types	[145]
Casein (acid-induced)	Acid-induced gelation	Crocin	In vitro: Pseudoplastic behavior, 58% release in 24 h, high loading with increased porosity	[147]
Selenium nanoparticles-casein-alginate	In situ green synthesis in hydrogel	Selenium nanoparticles	In vitro/in vivo: Targeted cancer therapy, sustained release, reduced systemic toxicity, eco-friendly synthesis	[149]
WPI-Amyloid-Gliadin	Amyloid fibril assembly and nanoparticle addition	Curcumin	In vitro: Enhanced stiffness, good encapsulation and protection of curcumin, stable and sustained release	[152]
WPI with TAs	pH-sensitive formulation, hydrogelation	TA derivatives	In vitro: Significant reduction in Hep-2 cell viability (up to 80% at 48 h), pH-controlled release profile	[163]
Collagen-Chitosan-PU	Composite 3D matrix formation	Ketorolac (NSAID)	In vitro: Controlled physiologic release, enhanced mechanical strength, antibacterial properties	[176]
Tilapia Collagen-Chitosan	Collagen/chitosan blend, pH-responsive	Anti-CEA and anti-PD-L1 nanobodies	In vitro: pH-dependent release, programmable delivery for targeted therapy	[177]
Elastin-like peptide hydrogel	Dynamic reversible physical interactions	Salvianolic acid B	In vitro/in vivo: Continuous release, cardiac repair, angiogenesis, minimally invasive	[193]
ELR	Click chemistry, MMP-sensitive domains	DOX	In vitro: 3D breast cancer cell culture; increased drug resistance over 2D models	[197]
Elastin-like polypeptide hydrogel	Tunable polymer, engineered control	DOX	In vitro/in vivo: Sustained local chemotherapy, improved outcome for malignant glioma	[200]
Engineered ELP hydrogel	Chemotherapeutic + PD-L1 inhibitor combination delivery	Chemo + PD-L1 antibody	In vivo: Enhanced immune response, increased tumor CD8+ T cell infiltration, potentiated immunotherapy	[201]

**Table 4:** Overview of some peptide and peptide–drug conjugate hydrogels used in cancer therapy and immunomodulation, highlighting the type of hydrogel system and the delivered therapeutic payload.

Peptide/Hydrogel System	Therapeutic Payload	References
Q_3_GPFSSTKT (Q_3_GT), Q_3_GSPTFSTK (Q_3_GK) and Ac-I_3_K- NH_2_	Mechanical + biochemical cues	[205]
Hydrogelator Nap-Phe-Phe-Arg-Arg-Lys-Ser-OH	AZD1152-HQPA (AZD, an Aurora kinase B inhibitor)	[208]
Cell penetrating peptide iRGD	Tubustecan prodrug (TT6) with either DOX or curcumin for combination chemotherapies	[209]
KKFKFEFEF (Lys-Lys-Phe-Lys-Phe-Glu-Phe-Glu-Phe)	Chemotherapeutic drug MTX	[210]
Curcumin-FFE-CS-EE peptide conjugate	Curcumin	[211]
C_16_-GNNQQNYKD-OH (C_16_-N) amphiphilic peptide	Losartan	[212]
FOE (FOFOFRFE) pH-responsive ionic-complementary octapeptide	DOX	[213]
Indomethacin -FFpYSV conjugated peptide derivative	Indomethacin and anticancer tripeptide (Tyr-SerVal, or YSV)	[214]
RADA16 peptide (Ac–RADARADARADARADA–NH2) and RADA16- R8 fusion peptide (Ac–RADARADARADARADA–GG–RRRRRRRR–NH2)	DOX	[215]
Peptide sequence of Phe-Phe-Arg-Phe-Lys (FFRFK), a segment of Arg-Gly-Glu (RGD) tripeptide anda cationic residue of Lys at C-terminus	Lonidamine + TPPS4	[216]
RGD-derived peptides (Gly-Phe-Phe-Tyr-Gly-Arg-Gly-Asp-His, GFFYGRGDH_n_)	DOX	[217]
Ac-(RADA)_4_-CONH_2_ peptide	DOX + Curcumin	[218]
K_2_(SL)_6_K_2_ (MDP) peptide	Cyclic dinucleotides (CDNs)	[220]
Naphthylacetic acid modified tetra-peptide of GFFY (Nap-GFFY)	OVA antigen	[221]
Poly(ethylene glycol)/poly(g-ethyl-L-glutamate) (PEG/PELG) polypeptide	DOX + IL-2 + IFN-γ	[222]
TpYCR peptide (mitochondria-targeting)	DOX	[223]

## Data Availability

No new data were created or analyzed in this study.

## Abbreviations

The following abbreviations are used in this manuscript:

3D	Three-dimensional
5-FU	5-fluorouracil
AA	Acrylic acid
AASF	Antheraea assamensis silk fibroin
AMPS	2-acrylamido-2-methylpropane sulfonic acid
BMSF	Bombyx mori silk fibroin
BSA	Bovine Serum Albumin
CA	Citric Acid
CBD	Cannabidiol
CDNs	Cyclic dinucleotides
CEA	Carcinoembryonic antigen
cGMP	Current Good Manufacturing Practice
CMC	Carboxymethyl cellulose
CRC	Colorectal cancer
DEX	Dexamethasone
DOX	Doxorubicin
ECM	Extracellular Matrix
EDC	1-ethyl-3-3-dimethylaminopropyl-carbodiimide
EGCG	Epigallocatechin Gallate
ELPs	Elastin-like polymers
ELRs	Elastin-like recombinamers
GAL	Glyceraldehyde
GelMA	Gelatin methacrylate
GLU	Glutaraldehyde
HEMA	2-hydroxyethyl methacrylate
HNSCC	Head and neck squamous cell carcinoma
HRP	Horseradish Peroxidase
LIPO-DOX	Doxorubicin-loaded liposomes
MI	Myocardial infarction
MRSA	Methicillin-resistant Staphylococcus aureus
MTX	Methotrexate
mTGase	Microbial transglutaminase
NBNAGA	N′-2-nitrobenzyl-N-acryloyl glycinamide
OVA	Ovalbumin
OXP	Oxaliplatin
PAAm	Polyacrylamide
PCL	Polycaprolactone
PD-L1	Programmed death-ligand 1
PDT	Photodynamic therapy
PEG	Polyethylene glycol
PGA	Polyglutamic acid
pI	Isoelectric Point
pTRG	Thermally responsive hydrogel
PTT	Photothermal therapy
PVA	Polyvinyl alcohol
ROS	Reactive oxygen species
SBMA	2-methacryloyloxy ethyl] dimethyl-3-sulfopropyl ammonium hydroxide
SF	Silk fibroin
SP	Soy protein
SPI	Soy protein isolate
TAs	Tannic acids
TGase	Transglutaminase
WPC	Whey protein concentrate
WPI	Whey protein isolate
γ-PGA	γ-polyglutamic acid
